# Causes of excess nonlymphoma death in 58 000 patients with DLBCL diagnosed during 1997 to 2020 and followed for up to 25 years

**DOI:** 10.1182/bloodadvances.2025017966

**Published:** 2026-03-06

**Authors:** Andrew Challenger, Paul McGale, Jake Probert, Aisling Barrett, John Broggio, Graham P. Collins, Kavya Kayiparambil Harish, Lorna Roden, Daniel Royston, Zhe Wang, Sarah C. Darby, David J. Cutter

**Affiliations:** 1 Nuffield Department of Population Health, University of Oxford, Oxford, United Kingdom; 2 Oxford Cancer and Haematology Centre, Oxford University Hospitals NHS Foundation Trust, Oxford, United Kingdom; 3 National Disease Registration Service, NHS England, Birmingham, United Kingdom; 4 Nuffield Division of Clinical Laboratory Sciences, Radcliffe Department of Medicine, University of Oxford, Oxford, United Kingdom; 5 Department of Pathology, Oxford University Hospitals NHS Foundation Trust, Oxford, United Kingdom

## Abstract

Survival in diffuse large B-cell lymphoma (DLBCL) has improved, raising interest in mortality from nonlymphoma causes in patients with DLBCL, which is known to be increased compared with the general population. We investigate excess mortality after DLBCL, quantifying both the absolute magnitude of the total nonlymphoma excess and the contributions of major disease groups. All 58 221 patients aged 18 to 79 years diagnosed with DLBCL as their first lymphoma in England during 1997 to 2020 were identified and followed for up to 25 years (median, 10.2 years). Absolute excess mortality rates (AERs) and standardized mortality ratios (SMRs) were estimated. A total of 30 596 patients died, 35.6% from lymphoma and 17.0% from other causes. The nonlymphoma mortality rate was 58% higher than that of the general population (SMR, 1.58; 95% confidence interval [CI], 1.55-1.61), with >100 excess nonlymphoma deaths per 10 000 person-years (AER, 103.8; 95% CI, 98.2-109.4). The nonlymphoma AER was substantial during the first year after diagnosis (AER, 369.1; 95% CI, 349.1-389.7). It subsequently fell but remained significantly elevated throughout follow-up, even beyond 10 years (AER, 93.7; 95% CI, 80.5-107.3). Disease groups with the biggest contributions to the AER were infection <1 year after diagnosis, constituting 29%; hematological causes excluding lymphoma (eg, leukemia) during years 1 to 4, 38%; and solid tumors thereafter, 31% in years 5 to 9 and 34% in years 10 to 25. Deaths from circulatory disease were elevated overall, but the SMR diminished in more recent calendar years of diagnosis. These insights may help guide treatment developments, interventions, and screening strategies toward reducing excess nonlymphoma deaths in the future.

## Key Points

• Death rates from nonlymphoma causes were higher in patients with DLBCL than the general population and stayed elevated even beyond 10 years.
• Infection was the largest contributor to the excess during the first year after diagnosis, and solid tumors were the largest beyond 5 years.

## Visual Abstract

**Figure:**
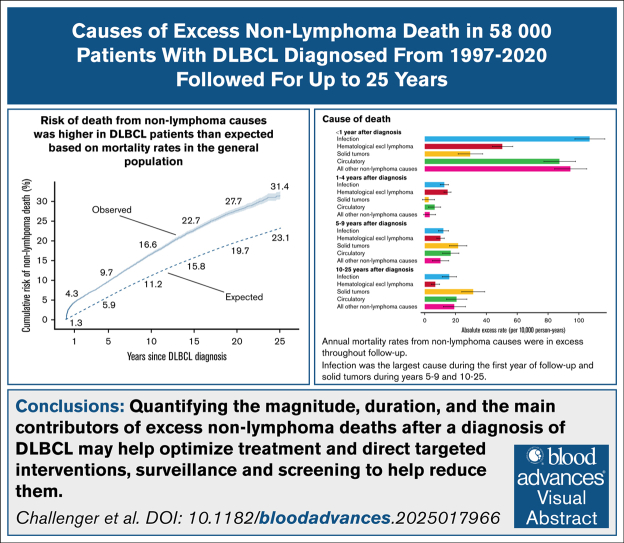

## Introduction

Non-Hodgkin lymphoma (NHL) is the seventh commonest cancer in the United Kingdom, accounting for ∼4% of new cancer cases during 2017 to 2019.^1^ Diffuse large B-cell lymphoma (DLBCL) is the commonest subtype of NHL with >5000 new diagnoses in the United Kingdom per year and accounting for ∼40% of NHL cases.^2^ An estimated 30 000 people currently living in the United Kingdom had a diagnosis of DLBCL during the last 10 years.^2^

For the last 2 decades, much research has focused on improving upon R-CHOP (rituximab, cyclophosphamide, doxorubicin, Oncovin [vincristine], and prednisolone)^3^ as the standard first-line treatment for DLBCL. Modest progress has been made in improving progression-free survival,^4^ and with current management, 5-year relative overall survival is ∼62%.^5^ In addition, it is now well recognized that patients with DLBCL die from nonlymphoma causes at a higher rate than the general population for many years after cure.^6^^,^^7^ Some clinical trials have used risk-adapted strategies to de-escalate initial treatment exposures while demonstrating noninferiority,^8^^,^^9^ but there has been little research directed specifically toward reducing excess nonlymphoma mortality in patients with DLBCL, either during initial treatment or in long-term survivors.

The aim of this study was to investigate excess mortality in a large population of adults diagnosed with DLBCL in England, quantifying both the absolute magnitude of the overall nonlymphoma excess and the contributions of major disease groups. We report findings in a manner intended both to help guide the long-term aftercare of survivors and to highlight areas in which clinical trials of specific interventions and studies examining surveillance strategies may be of benefit.

## Methods

### Study population

We received data from the National Cancer Registration and Analysis Service, which routinely registers all cancer diagnoses in England. Pseudonymized patient-level data on all adults (aged 18+ years) registered with their first lymphoma in England from 1 January 1997 to 31 December 2020 were provided. Patients with DLBCL were identified using morphological and diagnostic classification codes and histological descriptions (supplemental Methods A).

Data included gender, date of birth, quintiles of the index of multiple deprivation (an English measure of relative socioeconomic deprivation at small geographical area level),^10^ National Health Service (NHS) region, and date of DLBCL diagnosis. Tumor and treatment data, including Ann Arbor stage, route to diagnosis,^11^ Charlson Comorbidity Index,^12^ and use of systemic therapy and radiotherapy (RT), were available for some patients. Patients were followed until 31 December 2022, and for patients who died before this, date and certified causes of death using the International Classification of Diseases 9th/10th revision (ICD-9/10) were available. Underlying cause of death was assigned based on World Health Organization guidelines (supplemental Methods A). Individual codes were combined into clinically relevant groups (supplemental Table 1). Mortality rates by International Classification of Diseases code for the whole population of England, stratified by attained age, calendar year, gender, and deprivation quintile, were available from 1997 to 2022.

Patients were excluded if their index DLBCL was registered only using the death certificate or they had no follow-up; they were aged 80+ years at diagnosis; or they died, but no cause of death information was available (supplemental Figure 1). After investigation (supplemental Methods A), deaths from malignant neoplasms of unspecified/multiple sites were grouped with lymphoma deaths.

### Statistical methods

Patients entered the study on the date of their DLBCL diagnosis and were followed until the earliest of their date of death, their 85th birthday, or 31 December 2022. We censored at the 85th birthday because 5-year age-specific population mortality rates are only available until the age group 80 to 84 years. We therefore excluded patients aged 80+ years at diagnosis to allow all patients to have at least 5 years of follow-up.

The main statistical analysis had 4 components:

(1) Flexible parametric models^13^ were used to estimate cause-specific mortality rates from lymphoma and other causes. These models were then used to estimate the cumulative risk (CR) of death from lymphoma, adjusted for nonlymphoma deaths as competing events, and vice versa.
(2) Numbers of excess deaths (EDs), absolute excess mortality rates (AERs), and standardized mortality ratios (SMRs) were calculated to compare mortality rates in the DLBCL cohort with those of the general English population. EDs were calculated as follows: O - E, in which O and E are the numbers of observed and expected deaths from a given cause, respectively. To calculate E, the person-years at risk were subdivided into strata defined by attained age in 5-year groups, single calendar year, gender, and deprivation quintile. AERs were calculated as the number of EDs per 10 000 person-years and SMRs as O/E. Confidence intervals (CI) were obtained using the Poisson distribution.
(3) Observed and expected CR estimates were calculated to provide insight into the magnitude of the excess absolute risk. Follow-up was split into monthly intervals, and observed and expected deaths for each interval were calculated by the method described earlier. The CR was estimated nonparametrically using a method similar to the Aalen and Johansen estimator.^14^
(4) Poisson regression was used to describe the association between specific variables and cause-specific mortality. Rate ratios (RRs) were stratified by follow-up (split into regular intervals), age at and calendar year of diagnosis, gender, deprivation quintile, NHS region, and Charlson Comorbidity Index.

There were no missing values for 4 of the main variables used throughout (age at and calendar year of diagnosis, gender, and NHS region), and in the few cases in which deprivation quintile was missing, it was estimated using other geographical variables (supplemental Methods A). For the few analyses requiring other variables with appreciable numbers of missing values, subsets of the cohort with little/no missingness in these variables were used.

Median follow-up was estimated using the reverse Kaplan-Meier method.^15^ All *P* values were 2-sided. Tests for heterogeneity and trend were calculated using the likelihood ratio. All analyses were performed in R version 4.3.2. Further methodological details are provided in supplemental Methods B. A Shiny app was also developed to present the main results in an interactive format (https://livedataoxford.shinyapps.io/Excess_Mortality_After_DLBCL_App/).

## Results

### Characteristics of the cohort

A total of 58 221 patients with DLBCL were included in the study (Table 1). The median age at diagnosis was 66 years (interquartile range, 56-73), and 56.4% of patients were male. Systemic therapy was recorded for 80.9% and RT for 25.2%, and for both treatments, recorded use increased with calendar period. The median follow-up was 10.2 years (interquartile range, 6.2-15.0). At the end of follow-up, 27 625 patients (47.4%) were alive, 20 753 (35.6%) had died of lymphoma, and 9843 (17.0%) had died of nonlymphoma causes.

**Table 1.** **Characteristics of the DLBCL cohort**

Characteristic	Calendar period of diagnosis				All patients, n/N (%)
	1997-2005, n (%)	2006-2010, n (%)	2011-2015, n (%)	2016-2020, n (%)	
**Age at diagnosis, y**					
18-44	1 718 (13.0)	1 450 (11.3)	1 558 (9.8)	1 502 (9.2)	6 228 (10.7)
45-59	3 213 (24.3)	2 871 (22.5)	3 346 (21.0)	3 407 (20.8)	12 837 (22.0)
60-69	3 715 (28.2)	3 804 (29.8)	4 976 (31.3)	4 670 (28.6)	17 165 (29.5)
70-79	4 550 (34.5)	4 657 (36.4)	6 028 (37.9)	6 756 (41.4)	21 991 (37.8)
**Gender**					
Male	7 286 (55.2)	7 137 (55.8)	8 994 (56.5)	9 413 (57.6)	32 830 (56.4)
Female	5 910 (44.8)	5 645 (44.2)	6 914 (43.5)	6 922 (42.4)	25 391 (43.6)
**Deprivation quintile**					
<20% (least deprived)	2 873 (21.8)	2 714 (21.2)	3 453 (21.7)	3 409 (20.9)	12 449 (21.4)
20%-39%	2 914 (22.1)	2 819 (22.1)	3 446 (21.6)	3 528 (21.6)	12 707 (21.8)
40%-59%	2 674 (20.2)	2 607 (20.4)	3 242 (20.4)	3 379 (20.7)	11 902 (20.4)
60%-79%	2 428 (18.4)	2 412 (18.9)	3 082 (19.4)	3 109 (19.0)	11 031 (19.0)
≥80% (most deprived)	2 307 (17.5)	2 230 (17.4)	2 685 (16.9)	2 910 (17.8)	10 132 (17.4)
**NHS region**∗					
South West	1 656 (12.5)	1 388 (10.9)	1 807 (11.4)	2 010 (12.3)	6 861 (11.8)
North West	1 411 (10.7)	1 481 (11.6)	2 033 (12.8)	2 038 (12.5)	6 963 (12.0)
East of England	1 200 (9.1)	1 601 (12.5)	2 013 (12.6)	1 926 (11.8)	6 740 (11.6)
Midlands	2 814 (21.3)	2 686 (21.0)	3 066 (19.3)	3 182 (19.5)	11 748 (20.2)
South East	2 397 (18.2)	2 017 (15.8)	2 596 (16.3)	2 630 (16.1)	9 640 (16.5)
London	895 (6.8)	1 282 (10.0)	1 802 (11.3)	1 924 (11.8)	5 903 (10.1)
North East and Yorkshire	2 823 (21.4)	2 327 (18.2)	2 591 (16.3)	2 625 (16.0)	10 366 (17.8)
**Charlson Comorbidity Index**†					
0	0 (NA)	10 225 (80.0)	11 914 (74.9)	11 608 (71.1)	33 747 (75.0)
1	0 (NA)	1 319 (10.3)	1 997 (12.6)	2 183 (13.4)	5 499 (12.2)
2+	0 (NA)	1 238 (9.7)	1 992 (12.5)	2 527 (15.5)	5 757 (12.8)
Unknown	13 196 (NA)	0 (NA)	5 (NA)	17 (NA)	13 218 (NA)
**Route to diagnosis**‡					
Nonemergency	0 (NA)	8 269 (67.4)	10 272 (66.8)	6 432 (67.1)	24 973 (67.1)
Emergency	0 (NA)	3 996 (32.6)	5 108 (33.2)	3 149 (32.9)	12 253 (32.9)
Unknown	13 196 (NA)	517 (NA)	528 (NA)	6 754 (NA)	20 995 (NA)
**Systemic therapy**					
Systemic therapy recorded	8 430 (63.9)	10 101 (79.0)	13 956 (87.7)	14 641 (89.6)	47 128 (80.9)
None recorded	4 766 (36.1)	2 681 (21.0)	1 952 (12.3)	1 694 (10.4)	11 093 (19.1)
**RT**					
RT recorded	2 906 (22.0)	2 688 (21.0)	4 139 (26.0)	4 913 (30.1)	14 646 (25.2)
None recorded	10 290 (78.0)	10 094 (79.0)	11 769 (74.0)	11 422 (69.9)	43 575 (74.8)
**Combined therapy**					
Both therapies recorded	2 192 (16.6)	2 336 (18.3)	3 880 (24.4)	4 739 (29.0)	13 147 (22.6)
Only systemic therapy recorded	6 238 (47.3)	7 765 (60.7)	10 076 (63.3)	9 902 (60.6)	33 981 (58.3)
Only RT recorded	714 (5.4)	352 (2.8)	259 (1.6)	174 (1.1)	1 499 (2.6)
None recorded	4 052 (30.7)	2 329 (18.2)	1 693 (10.7)	1 520 (9.3)	9 594 (16.5)
**Duration of follow-up, y**					
<1	3 895 (29.5)	3 286 (25.7)	3 711 (23.3)	3 789 (23.2)	14 681 (25.2)
1-4	2 499 (18.9)	2 122 (16.6)	2 614 (16.4)	8 498 (52.0)	15 733 (27.0)
5-9	1 775 (13.5)	1 941 (15.2)	7 010 (44.1)	4 048 (24.8)	14 774 (25.4)
10-25	5 027 (38.1)	5 433 (42.5)	2 573 (16.2)	0 (0.0)	13 033 (22.4)
**Vital status at end of follow-up**					
Alive	4 018 (30.4)	5 334 (41.7)	8 117 (51.0)	10 156 (62.2)	27 625 (47.4)
Died of lymphoma	6 004 (45.5)	4 777 (37.4)	5 372 (33.8)	4 600 (28.2)	20 753 (35.6)
Died of causes excluding lymphoma	3 174 (24.1)	2 671 (20.9)	2 419 (15.2)	1 579 (9.7)	9 843 (17.0)
Infection	353 (2.7)	321 (2.5)	330 (2.1)	413 (2.5)	1 417 (2.4)
Hematological excluding lymphoma	167 (1.3)	217 (1.7)	218 (1.4)	134 (0.8)	736 (1.3)
Solid tumors	851 (6.4)	700 (5.5)	617 (3.9)	344 (2.1)	2 512 (4.3)
Circulatory	1 044 (7.9)	689 (5.4)	541 (3.4)	281 (1.7)	2 555 (4.4)
All other nonlymphoma causes	759 (5.8)	744 (5.8)	713 (4.5)	407 (2.5)	2 623 (4.5)
Total	13 196 (100.0)	12 782 (100.0)	15 908 (100.0)	16 335 (100.0)	58 221 (100.0)

Unknown values do not contribute toward column percentages. Further information on cohort characteristics is given in supplemental Table 2.

NA, not applicable.

∗ NHS region is used as a stratification variable in some analyses, because levels of systemic therapy and RT vary by region.

† Data on Charlson Comorbidity Index were unavailable in patients diagnosed before 2006.

‡ Data on route to diagnosis were unavailable in patients diagnosed before 2006 or after 2018.

### Deaths due to lymphoma and nonlymphoma

**Figure 1.**
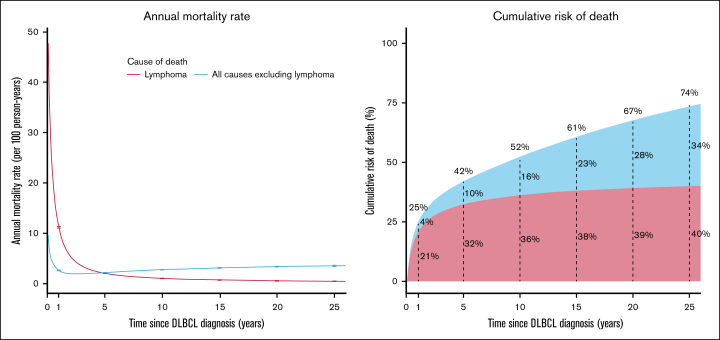
**Mortality rate and CR of death for the DLBCL cohort, by time since diagnosis and cause of death.** Error bars for mortality rates indicate 95% CIs. The estimates of CR for death from lymphoma are adjusted for nonlymphoma causes as a competing risk, and vice versa, so that the 2 risks sum to the CR of death from any cause.

The lymphoma-specific mortality rate was very high in the first month after diagnosis (48 deaths per 100 person-years) but then deceased rapidly over the first 3 years and more slowly thereafter (Figure 1). The nonlymphoma mortality rate was also high in the first month (10 deaths per 100 person-years) and then decreased but, in contrast to lymphoma-specific mortality, began to increase from ∼2 years after diagnosis and exceeded the lymphoma-specific rate after ∼5 years. This pattern was similar across calendar periods of diagnosis, with the 5-year CR of nonlymphoma death consistently ∼10% (supplemental Figure 2). The CR of lymphoma death was greater than that of nonlymphoma death throughout follow-up but decreased with diagnostic period (5-year CR, 39% in 1997-2005 vs 29% in 2016-2020), so that nonlymphoma mortality constituted a greater proportion of all-cause mortality in more recent periods (∼1/5 in 1997-2005 vs 1/4 in 2016-2020 at 5 years).

**Figure 2.**
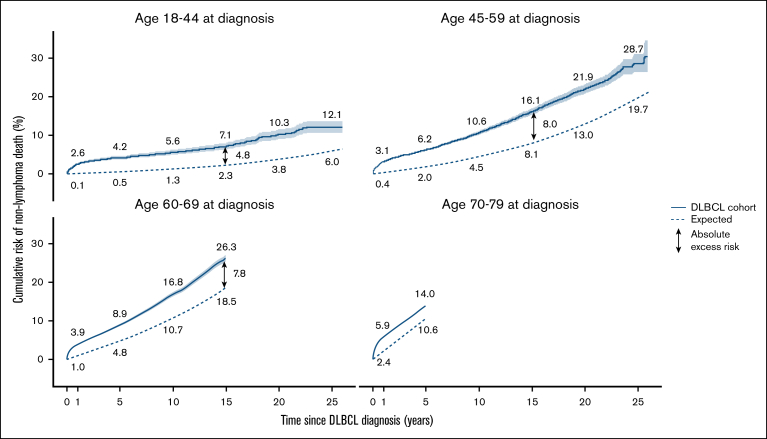
**CR of death from all causes other than lymphoma for the DLBCL cohort, by time since diagnosis and age at diagnosis.** Solid lines represent the observed risk for DLBCL cohort and dashed lines the risk that would be expected based on mortality rates for nonlymphoma causes for the English population, accounting for calendar period, attained age, gender, and deprivation quintile. Ribbons indicate 95% CI. Both observed and expected CR estimates are adjusted for death from lymphoma as a competing risk. For each age at diagnosis group, follow-up is stopped for the whole group when the oldest patient in the group turns 85 years to ensure the CR estimates are representative of the whole group.

Overall, the nonlymphoma mortality rate was nearly 60% higher in patients with DLBCL than in the English general population (SMR, 1.58; 95% CI, 1.55-1.61; supplemental Table 3), and there was little evidence of a decrease in the SMR in more recent calendar periods of diagnosis, either during the first year or in later follow-up periods (supplemental Figures 3-5). Compared with the general population, there were ∼100 excess nonlymphoma deaths (ENLDs) per year for every 10 000 patients with DLBCL (AER, 103.8; 95% CI, 98.2-109.4; Table 2). This AER was largest during the first year after diagnosis (AER, 369.1; 95% CI, 349.1-389.7), falling to 39.3 (95% CI, 31.7-47.0) during 1 to 4 years, then increasing to 70.7 (95% CI, 60.7-81.0) during 5 to 9 years and 93.7 (95% CI, 80.5-107.3) during 10 to 25 years, thus remaining significantly elevated throughout follow-up. The observed 15-year CR of nonlymphoma death was 4.8% higher than expected (ie, 7.1% vs 2.3%) in patients aged 18 to 44 years at diagnosis; 8.0% for those aged 45 to 59 years; and 7.8% for those aged 60 to 69 years (Figure 2). In patients aged 70 to 79 years, the AER at 5 years was 3.4%, largely due to a high mortality rate in the first year after diagnosis. There was no evidence that patients aged 70 to 79 years at diagnosis experienced an excess nonlymphoma mortality during years 1 to 4 (supplemental Figure 4). During the first year after a DLBCL diagnosis, both the lymphoma and the nonlymphoma mortality rates in patients diagnosed after an emergency presentation were higher than in those presenting in other ways for all ages combined (lymphoma [RR, 3.18; 95% CI, 3.03-3.33]; other causes [RR, 2.19; 95% CI, 1.96-2.44]; supplemental Figure 6) and for younger and older patients considered separately (supplemental Figure 7).

**Table 2.** **AERs per 10 000 person-years for the DLBCL cohort by cause of death and time since diagnosis**

Time since diagnosis	<1 year		1-4 years		5-9 years		10-25 years		Overall	
Cause of death	Obs	AER (95% CI)	Obs	AER (95% CI)	Obs	AER (95% CI)	Obs	AER (95% CI)	Obs	AER (95% CI)
All causes	14 681	2860.6 (2812.1-2909.8)	9468	499.2 (485.6-513.0)	4072	211.7 (199.1-224.5)	2375	186.1 (170.8-201.8)	30 596	691.4 (681.6-701.2)
Lymphoma	12 161	NA	6521	NA	1460	NA	611	NA	20 753	NA
**All causes excluding lymphoma**	2 520	369.1 (349.1-389.7)	2947	39.3 (31.7-47.0)	2612	70.7 (60.7-81.0)	1764	93.7 (80.5-107.3)	9 843	103.8 (98.2-109.4)
COVID-19∗	127	24.5 **(**20.1-29.4)	112	4.6 **(**3.2-6.2)	83	3.2 **(**1.5-5.2)	84	5.7 **(**3.0-8.9)	406	7.2 **(**6.1-8.4)
Pneumonia and other respiratory infections	144	23.0 **(**18.4-28.3)	144	2.4 **(**0.8-4.3)	130	4.1 **(**1.9-6.5)	102	8.0 **(**5.0-11.5)	520	6.8 **(**5.5-8.1)
Other infections	297	59.6 **(**52.8-66.9)	102	5.6 **(**4.2-7.1)	67	4.8 **(**3.3-6.6)	25	2.1 **(**0.7-4.1)	491	12.3 **(**11.1-13.6)
**All infections**	568	107.1 (97.7-117.1)	358	12.6 (10.1-15.4)	280	12.1 (8.8-15.6)	211	15.9 (11.4-20.8)	1417	26.2 (24.1-28.4)
Acute leukemia	44	8.0 **(**5.5-11.1)	111	6.8 **(**5.4-8.4)	55	4.3 **(**2.9-6.0)	32	3.9 **(**2.2-6.0)	242	5.7 **(**4.9-6.6)
Chronic leukemia	34	6.5 **(**4.4-9.3)	31	1.7 **(**1.0-2.6)	15	0.9 **(**0.2-1.9)	7	0.5 **(**−0.1 to 1.7)	87	1.9 **(**1.4-2.5)
MDS and other hematological neoplasms	109	21.9 **(**17.9-26.6)	64	4.0 **(**2.9-5.3)	34	2.7 **(**1.7-4.1)	15	1.7 **(**0.6-3.3)	222	5.7 **(**4.9-6.6)
Myeloma	20	3.0 **(**1.4-5.3)	24	0.5 **(**−0.1 to 1.4)	15	0.2 **(**−0.5 to 1.2)	8	0.0 **(**−0.8 to 1.2)	67	0.7 **(**0.2-1.2)
**All hematological (excluding lymphoma)**†	262	50.4 (44.1-57.3)	261	14.8 (12.6-17.2)	144	10.2 (7.9-12.7)	69	6.8 (4.3-9.7)	736	17.0 (15.5-18.6)
Colorectal cancer	33	1.2 **(**−0.9 to 4.0)	87	0.2 **(**−1.1 to 1.6)	80	1.5 **(**−0.1 to 3.5)	57	2.8 **(**0.6-5.6)	257	1.2 **(**0.3-2.1)
Pancreas cancer	26	2.1 **(**0.2-4.6)	36	−1.1 **(**−1.8 to −0.1)	47	0.7 **(**−0.5 to 2.3)	53	4.5 **(**2.3-7.1)	162	0.9 **(**0.2-1.6)
**All gastrointestinal cancers**†	143	12.5 (7.8-17.7)	256	−0.3 (−2.4 to 2.1)	254	5.4 (2.3-8.7)	199	12.0 (7.7-16.8)	852	5.3 (3.7-7.0)
Head and neck cancers	8	0.3 **(**−0.6 to 1.9)	27	0.5 **(**−0.1 to 1.4)	29	1.4 **(**0.5-2.7)	20	1.6 **(**0.4-3.4)	84	0.9 **(**0.5-1.5)
Lung cancer	58	−2.0 **(**−4.9 to 1.5)	212	0.7 **(**−1.3 to 2.9)	233	8.3 **(**5.4-11.6)	152	10.0 **(**6.2-14.2)	655	4.1 **(**2.7-5.6)
Melanoma of skin	2	−0.3 **(**−0.7 to 0.7)	26	1.0 **(**0.4-1.9)	22	1.3 **(**0.5-2.4)	11	0.8 **(**−0.0 to 2.2)	61	0.9 **(**0.5-1.4)
Female breast cancer‡	17	1.0 **(**−2.4 to 5.8)	25	−3.3 **(**−4.8 to −1.4)	38	0.8 **(**−1.7 to 3.9)	32	4.0 **(**0.4-8.7)	112	−0.3 **(**−1.5 to 1.2)
Prostate cancer‡	13	−2.2 **(**−4.4 to 1.2)	50	−1.8 **(**−3.5 to 0.2)	59	1.5 **(**−1.1 to 4.6)	31	0.0 **(**−2.9 to 3.8)	153	−0.6 **(**−1.8 to 0.7)
Kidney cancer	15	1.7 **(**0.3-3.7)	20	−0.1 **(**−0.6 to 0.7)	14	−0.2 **(**−0.9 to 0.7)	13	0.5 **(**−0.5 to 1.9)	62	0.2 **(**−0.2 to 0.7)
Bladder (and other urothelial) cancer	6	−0.6 **(**−1.4 to 0.8)	27	−0.2 **(**−0.9 to 0.6)	36	1.2 **(**0.1-2.6)	29	2.2 **(**0.7-4.3)	98	0.5 **(**0.0-1.1)
**All solid tumors**†	385	29.5 (21.8-37.8)	787	2.4 (−1.4 to 6.5)	791	21.6 (16.1-27.4)	549	31.4 (24.1-39.1)	2 512	16.8 (14.0-19.6)
Ischemic heart disease	350	47.6 **(**40.3-55.5)	394	2.8 **(**0.1-5.7)	324	6.8 **(**3.3-10.5)	180	5.4 **(**1.3-10.0)	1 248	10.6 **(**8.7-12.7)
Cardiomyopathy and congestive heart failure	54	8.4 **(**5.7-11.8)	80	2.4 **(**1.3-3.8)	87	5.0 **(**3.3-7.1)	77	8.8 **(**6.2-11.9)	298	5.1 **(**4.2-6.2)
Valvular heart disease	23	3.3 **(**1.6-5.7)	27	0.1 **(**−0.5 to 1.0)	43	2.2 **(**1.0-3.7)	28	2.4 **(**0.9-4.4)	121	1.6 **(**1.0-2.2)
Arrhythmias	19	3.1 **(**1.6-5.3)	32	1.1 **(**0.4-2.0)	25	0.9 **(**0.0-2.1)	28	2.8 **(**1.3-4.8)	104	1.6 **(**1.1-2.3)
**All cardiac**†	464	65.2 (56.8-74.3)	549	6.5 (3.3-10.0)	493	15.1 (10.8-19.7)	326	20.1 (14.6-26.2)	1 832	19.5 (17.2-22.0)
Cerebrovascular	89	8.8 **(**5.2-13.0)	157	0.2 **(**−1.5 to 2.1)	134	1.6 **(**−0.6 to 4.1)	79	2.1 **(**−0.5 to 5.3)	459	2.1 **(**1.0-3.4)
**All circulatory**†	646	87.5 (77.5-98.1)	786	6.4 (2.5-10.4)	692	16.8 (11.6-22.2)	431	20.6 (14.2-27.5)	2 555	23.1 (20.3-26.0)
COPD and asthma	108	12.2 **(**8.2-16.8)	131	−2.0 **(**−3.5 to −0.2)	141	1.5 **(**−0.8 to 4.1)	78	0.2 **(**−2.4 to 3.3)	458	1.4 **(**0.2-2.6)
Other respiratory diseases	72	12.1 **(**8.9-16.0)	93	3.4 **(**2.2-4.9)	64	2.8 **(**1.3-4.6)	40	2.9 **(**1.1-5.3)	269	4.4 **(**3.5-5.3)
Digestive system disease	213	36.1 **(**30.4-42.4)	172	3.6 **(**1.9-5.6)	129	3.4 **(**1.3-5.9)	99	6.4 **(**3.4-9.9)	613	8.6 **(**7.2-10.0)
External causes (excluding suicide)	73	11.8 **(**8.5-15.6)	73	1.4 **(**0.3-2.7)	50	0.6 **(**−0.7 to 2.3)	41	2.1 **(**0.2-4.5)	237	2.8 **(**1.9-3.7)
All causes other than hematological, solid tumor, circulatory and infection†	659	94.6 (84.5-105.4)	755	3.1 (−0.7 to 7.1)	705	10.2 (5.0-15.6)	504	19.1 (12.2-26.5)	2 623	20.7 (17.8-23.6)

AERs were calculated by comparing to all England rates accounting for the same calendar period, attained age, gender, and deprivation quintile.

COPD, chronic obstructive pulmonary disease; MDS, myelodysplastic syndrome; NA, not applicable; Obs, observed deaths.

∗ Results for COVID-19 were based on follow-up data from 2020 onward.

† Only a subset of the individual causes of death are shown on the table, which is the reason for the observed deaths not summing to the total number in the major group. For results with all individual causes, see supplemental Table 3.

‡ For causes that only apply to 1 gender, calculations were performed using the subcohort corresponding to that gender.

### Deaths due to infection

**Figure 3.**
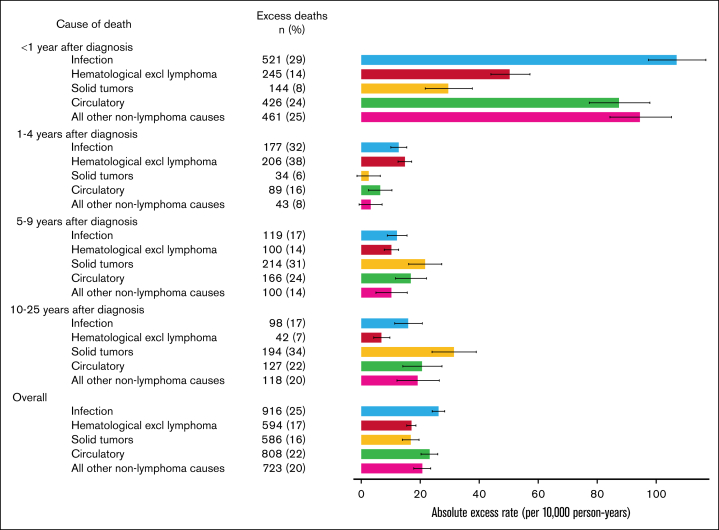
**ENLDs and AERs for the major causes of nonlymphoma death in the DLBCL cohort, by time since diagnosis.** Error bars indicate 95% CIs. EDs and AERs were calculated by comparing to all England rates accounting for attained age, calendar year, gender, and deprivation quintile.

Patients with DLBCL experienced significant excess mortality from infection during all follow-up periods, including 10+ years after diagnosis (Table 2). Infection constituted 29%, 32%, 17%, and 17% of ENLDs during years <1, 1 to 4, 5 to 9, and 10 to 25, respectively (Figure 3). Deaths from infection were the commonest cause of ENLDs both during the first year after diagnosis and overall. Considering only patients diagnosed from 2011, infection was also the commonest nonlymphoma cause during years 1 to 4 (supplemental Figure 8).

During the first year after diagnosis, infection constituted a larger percentage of ENLDs in younger patients than older patients (61% in ages 18-44 years vs 23% in ages 70-79 years; supplemental Figure 9), in men than women (32% vs 25%), and in patients diagnosed more recently (44% in 2016-2020 vs 21% in 1997-2005; supplemental Figure 10).

### Deaths due to hematological causes, excluding lymphomas

Patients with DLBCL experienced significant excess mortality from hematological causes excluding lymphomas (referred to as “hematological causes” hereafter) during all follow-up periods, constituting 14%, 38%, 14%, and 7% of ENLDs during years <1, 1 to 4, 5 to 9, and 10 to 25, respectively (Figure 3).

After the first year, hematological causes constituted a slightly larger percentage of ENLDs in younger patients than older (24% in ages 18-44 years at diagnosis vs 18% in ages 60-69 years; supplemental Figure 11) and in men than women (21% vs 16%). Over all age groups, there was no evidence that the SMR during years 1 to 4 changed with calendar period of diagnosis (SMR, 4.5 in 1997-2005 vs 5.2 in 2011-2015; *P*_trend_ = .520; supplemental Figure 4), but for patients aged 70 to 79 years, there was evidence of an increase in the SMR in more recent periods (SMR, 1.8 in 1997-2005 vs 3.7 in 2011-2015; *P*_trend_ = .015).

Deaths from hematological causes were the commonest cause of ENLDs during years 1 to 4 after diagnosis, and the specific causes with the largest AERs were acute leukemia (AER, 6.8; 95% CI, 5.4-8.4) and myelodysplastic syndrome and other hematological neoplasms (AER, 4.0; 95% CI, 2.9-5.3; Table 2).

### Deaths due to solid tumors

Patients with DLBCL experienced significant excess mortality from solid tumors during years <1, 5 to 9, and 10 to 25 after diagnosis, constituting 8%, 31%, and 34% of ENLDs during these follow-up periods, respectively (Figure 3). Deaths from solid tumors were not significantly elevated during years 1 to 4 but were the commonest cause of ENLDs from 5 years after diagnosis.

Solid tumors constituted a higher percentage of ENLDs in older patients than younger (supplemental Figures 9 and 11). There was no evidence of a reduction in the long-term SMR from solid tumors (SMR, 1.5 during years 5-11 in 1997-2005 vs 1.4 in 2006-2010; *P*_trend_ = .590; supplemental Figure 5).

During years 5 to 9 after diagnosis, the solid tumor causes with the largest AERs were lung cancer (AER, 8.3; 95% CI, 5.4-11.6) and gastrointestinal cancers (AER, 5.4; 95% CI, 2.3-8.7; Table 2). During years 10 to 25, these were gastrointestinal cancers (AER, 12.0; 95% CI, 7.7-16.8), lung cancer (AER, 10.0; 95% CI, 6.2-14.2), female breast cancer (AER, 4.0; 95% CI, 0.4-8.7), and bladder cancer (AER, 2.2; 95% CI, 0.7-4.3).

**Figure 4.**
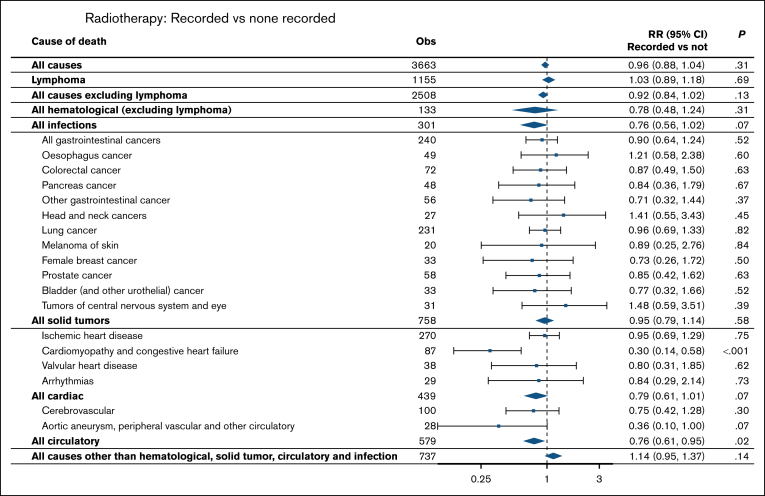
**RRs by recorded use of RT for the 45 025 patients with DLBCL diagnosed from 2006 onward, from 5 years after diagnosis.** Individual causes are shown for individual types of solid tumors and circulatory diseases if there were at least 20 observed deaths. RRs are stratified by age at diagnosis, calendar period of diagnosis, gender, deprivation quintile, NHS region, and Charlson Comorbidity Index, using the categories in Table 1, as well as the time since diagnosis in yearly intervals. Obs, observed deaths.

There was no significant association between the recorded use of RT and the risk of death from all solid tumors in patients with DLBCL who survived the first 5 years after diagnosis (RR = 0.96; 95% CI, 0.88-1.04), nor were there any significant associations with any individual types of solid tumor (Figure 4).

### Deaths due to circulatory disease

Patients with DLBCL experienced significant excess mortality from circulatory disease during all follow-up periods, constituting 24%, 16%, 24%, and 22% of ENLDs at years <1, 1 to 4, 5 to 9, and 10 to 25, respectively (Figure 3).

During the first year after diagnosis, circulatory disease constituted a larger percentage of ENLDs in older than younger patients (31% in ages 70-79 years vs 6% in ages 18-44 years) and in men than women (25% vs 21%; supplemental Figure 9). Circulatory disease constituted a smaller percentage of first-year ENLDs in more recent calendar periods (36% in 1997-2005 vs 12% in 2016-2020; supplemental Figure 10), in line with a significant reduction in the first-year SMR for circulatory disease in recent calendar periods (SMR, 3.4 in 1997-2005 vs 2.2 in 2016-2020; *P*_trend_ < .001; supplemental Figure 3). In patients diagnosed from 2011, there was no evidence of mortality excess from circulatory disease during years 1 to 4 after diagnosis (supplemental Figures 4 and 8), and the absolute excess CRs were smaller than in earlier periods (supplemental Figure 12).

Over all follow-up, the circulatory causes with the largest AERs were ischemic heart disease (AER, 10.6; 95% CI, 8.7-12.7), cardiomyopathy and congestive heart failure (AER, 5.1; 95% CI, 4.2-6.2), and cerebrovascular (AER, 2.1; 95% CI, 1.0-3.4; Table 2).

There was a strong negative association between the recorded use of RT and the risk of death from cardiomyopathy and congestive heart failure (RR = 0.30; 95% CI, 0.14-0.58; *P* < .001) but no association for ischemic heart disease (RR = 0.95; 95% CI, 0.69-1.29; *P* = .75) (Figure 4).

### Deaths due to all other nonlymphoma causes

Patients with DLBCL experienced significant excess mortality from all causes other than those detailed earlier (infection, hematological, solid tumor, and circulatory) during years <1, 4 to 9, and 10 to 25 after diagnosis, constituting 25%, 14%, and 20% of ENLDs during these follow-up periods, respectively (Figure 3). Over all follow-up, the causes with the largest AERs from this group were digestive system disease (AER = 8.6, 95% CI 7.2-10.0) and respiratory diseases (excluding asthma and chronic obstructive pulmonary disease; AER, 4.4; 95% CI, 3.5-5.3), which were elevated during every follow-up period (Table 2).

## Discussion

This is, to our knowledge, the largest population-based study assessing short- and long-term excess nonlymphoma mortality after a diagnosis of DLBCL. The size of the study and the greater length of follow-up compared to recent similar studies^8^^,^^9^ provide insight into the contributions of the major disease groups to the total nonlymphoma AER and how they vary with time after DLBCL diagnosis.

Lymphoma is the predominant cause of death in the first years after a DLBCL diagnosis, with nonlymphoma becoming the predominant cause after ∼5 years. Despite this changeover, lymphoma remains the larger contributor to the CR of death even at 25 years, suggesting that improved anticancer therapy for DLBCL remains an important goal. First-year mortality rates from both lymphoma and nonlymphoma causes were higher in patients presenting as an emergency rather than via routine referral pathways, suggesting also that diagnosis before patients with DLBCL become acutely unwell may help reduce early excess mortality.

Deaths from nonlymphoma causes occurred at a rate 60% higher than in the general population, with ∼100 ENLDs per 10 000 person-years. ENLDs in this cohort are, by definition, associated with a prior diagnosis of DLBCL and are therefore lymphoma related in some way, despite the underlying cause of death not being recorded as lymphoma. The AER was particularly large during the first year after diagnosis, which is consistent with other reports.^8^^,^^9^ However, our study, with its long follow-up, is, to our knowledge, the first to report that nonlymphoma mortality remained significantly elevated beyond 10 years after DLBCL diagnosis. Moreover, there was little evidence of a reduction in nonlymphoma excess mortality in recent years despite increasing recognition of the long-term adverse effects of lymphoma treatments. This should motivate research into reducing the toxicity of current DLBCL management, with the aim of reducing this excess in the future.

Infection was the commonest cause of ENLDs during the first year after diagnosis, particularly so in younger patients, men, and those diagnosed more recently. Previous studies have demonstrated that the risk remains elevated during the period of 5 to 10 years from diagnosis,^8^^,^^16^ but this study is, to our knowledge, the first to demonstrate an elevated risk beyond 10 years. DLBCL is known to result in immunological dysfunction, reducing normal B-cell numbers and function,^17^ and DLBCL treatments including chemotherapy, rituximab, corticosteroids, and RT are all recognized to have detrimental effects on immune networks.^18^ In the TEAMM trial in newly diagnosed myeloma, prophylactic levofloxacin during the first 12 weeks of therapy reduced febrile episodes and deaths compared with placebo,^19^ and in a recent cohort study of older patients with DLCBL (aged >70 years), the prescription of primary quinolone prophylaxis was associated with a reduction in infection-related admission.^20^ However, that study did not find a reduction in infection-related death, and a meta-analysis of interventions to reduce infection in hematological malignancies showed no improvement in survival.^21^ Although growth factor support and antimicrobial prophylaxis are used selectively in DLBCL, there has been little investigation into how best to direct their use.^22^ Whether patients at higher risk could be targeted with enhanced antimicrobial prophylaxis to reduce early EDs from infection remains unknown and would require randomized studies. Vaccination of patients with DLBCL for infections including pneumonia, influenza, and COVID-19 are recommended as part of standard practice. In the United Kingdom, uptake of the COVID-19 vaccine was reported to be higher in patients with blood cancer than in the general population,^23^ but even in recognized high-risk groups, adherence to vaccination in the United Kingdom did not reach the World Health Organization target.^24^ Improved awareness of longer-term risks in DLBCL survivors and greater adherence to vaccination recommendations for those at high risk could potentially help reduce EDs from infection. Which pathogens underlie EDs from infection in the longer term and the possibility of interventions to prevent these have not been systemically investigated. Reducing this risk could have a substantial effect on the long-term health and life expectancy of DLBCL survivors.

Deaths from hematological causes (excluding lymphoma) were the commonest cause of ENLDs in years 1 to 4, primarily due to acute leukemias and myelodysplastic syndromes, which is consistent with findings from other studies.^9^ Compared to de novo acute leukemia, these DLBCL/therapy-related cases have been shown to have a poorer prognosis.^25^ Some studies have suggested that acute myeloid leukemia after DLBCL has been more common in the rituximab era,^26^ but a causative link has not been established.

Deaths from solid tumors were elevated in the first year after diagnosis and again during periods 5 to 9 and 10 to 25 years after diagnosis, during which they were the commonest cause of ENLDs. Although 1 study of patients with DLBCL with a median follow-up 5.3 years did not find an excess mortality of solid tumors in the period 5+ years since diagnosis,^7^ 2 other studies of the incidence of second primary cancers, both with a median follow-up of over 10 years, found elevated risks throughout 20 years of follow-up.^27^^,^^28^ Such results suggest that efforts to minimize therapeutic exposures known to increase second cancer risks, as well as other exposures such as smoking,^28^ may help reduce long-term EDs from solid tumors. Lung cancer was among the largest causes of ED from any solid tumor, and it may be that individuals with additional risk factors (eg, smokers) are at high enough risk to warrant lung cancer screening via national programs.^29^ We found no association between recorded use of RT and death from solid tumors, consistent with other studies on second cancer incidence after DLBCL.^27^^,^^28^

Deaths from circulatory disease were significantly elevated throughout follow-up. The early increase is consistent with other studies reporting mortality during the first 5 years after diagnosis^6^^,^^7^ and also with a systematic review and meta-analysis of long-term cardiovascular risk.^30^ When we considered only those diagnosed more recently (2011 onward), we found no mortality excess from circulatory disease at 1 to 4 years and little excess up to 10 years, suggesting that the excess may have has decreased in recent years. It is encouraging that changes in management, including reduced anthracycline exposure, may have reduced cardiovascular risks from treatment. We found that recorded use of RT was not associated with an increased risk of any type of heart disease and, in fact, was associated with a lower risk of death from cardiomyopathy and heart failure. This is in agreement with previous studies^31^^,^^32^ and is likely due to patients treated with chemotherapy alone generally receiving a higher anthracycline exposure.

Deaths from all causes other than infection, hematological, solid tumor, or circulatory followed a similar temporal pattern to solid tumors, being significantly elevated in the first year after diagnosis and then again beyond 5 years. The causes with greatest AERs were respiratory diseases (excluding chronic obstructive pulmonary disease and asthma) and digestive diseases. Although deaths from respiratory disease have previously been shown to be elevated up to 5 years after diagnosis,^8^^,^^9^ our study is, to our knowledge, the first to demonstrate an excess risk in the longer term.

Our study has several strengths. The study is population based, so our results are widely representative and generalizable to the management of DLBCL in England over the last 25 years. In contrast to some other studies, our study encompasses a broad age spectrum (aged 18-79 years at diagnosis), includes patients treated with RT,^7^ is not restricted to patients surviving 5 years,^28^ and has a median follow-up of >10 years. In addition, the focus on the absolute magnitude of the nonlymphoma mortality excess provides researchers with appropriate information on the specific causes to target with preventative interventions, both at diagnosis/initial treatment and during each follow-up period.

Our study does, however, have limitations. First, it relies on the underlying cause of death as recorded on death certificates. It is recognized that death certificates are not always appropriately completed,^33^ and there may be substantial discrepancies between diagnoses given on death certificates compared with those after autopsy.^34^ However, we compare certified causes of death in patients with DLBCL to those of the English population, and they have all been collected and coded in the same way. Therefore, although the precise causes of death may at times be questioned, observed excesses are likely to be real. Second, due to its retrospective nature, our study is not well suited to establishing causality. However, the primary aim is to describe patterns of excess mortality, rather than demonstrate causation, so this does not substantially limit our research. Finally, lack of treatment information carries certain limitations. For example, a likely lack of completeness of recording of RT use in the earlier diagnostic periods may have diluted and reduced the significance of any true associations between RT use and various mortality end points. Similarly, although we lack data completeness on the receipt of chemotherapy, the use of CHOP or R-CHOP (from ∼2006) has been the standard first-line therapy for DLBCL and it is likely that most long-term survivors will have been exposed either to the chemotherapy agents contained within this regimen, namely cyclophosphamide, doxorubicin, vincristine and prednisolone, with or without rituximab, or to other similarly intensive chemotherapy regimens. Some of these agents undoubtedly contribute to a proportion of the ENLDs seen within our cohort, especially acute leukemia, myelodysplasia,^35^ cardiomyopathy and heart failure due to doxorubicin,^36^ and bladder cancer due to cyclophosphamide.^37^ The quantity and quality of data that are routinely collected in England are both improving, and so, in future, we hope that more detailed information, for example, on treatment and lymphoma recurrence, will be usable in population-based studies of DLBCL to identify more specific associations between treatments and long-term outcomes.

In conclusion, this is, to our knowledge, the largest study published on excess nonlymphoma mortality after a diagnosis of DLBCL. It provides valuable insights into the dominant causes of ENLDs and how these evolve with time since diagnosis and, to a lesser extent, calendar year of diagnosis. Our study highlights specific areas in which outcomes may be improved, including prevention of deaths from infection, cardiac causes, lung cancer, and acute leukemia. Optimization of treatment strategies for DLBCL to reduce exposures with proven risk are a first step but often require randomized trials. Specific interventional studies aimed at secondary risk reduction (eg, promotion of vaccination) and studies of surveillance strategies (eg, cardiovascular and cancer screening) may be of benefit in the period after treatment. Additionally, general health promotion (for example smoking cessation) and tailored survivorship plans for patients will empower individuals in reducing their own risks. Together, these measures may all help reduce excess nonlymphoma mortality in patients diagnosed with DLBCL.

Conflict-of-interest disclosure: A.C., P.M., J.P., K.K.H., L.R., Z.W., S.C.D., and D.J.C. report research funding from Cancer Research UK. G.P.C. reports honoraria for speaker and/or advisory work from Roche, AstraZeneca, Takeda, AbbVie, Genmab, BeOne, Sobi, Incyte, and SecuraBio; and research support from Pfizer, BeOne, AstraZeneca, Amgen, and Bristol Myers Squibb. The remaining authors declare no competing financial interests.

## Supplementary Material

Supplemental Methods, References, Tables, and Figures
