# Supplementary material for: Causes of excess nonlymphoma death in 58 000 patients with DLBCL diagnosed during 1997 to 2020 and followed for up to 25 years
Source: Blood Adv. 2026 Mar 6;10(9):3243–54. doi: 10.1182/bloodadvances.2025017966 (PMC13158759; doi:10.1182/bloodadvances.2025017966)
Supplement: Supplemental Methods, References, Tables, and Figures [file BLOODA_ADV-2025-017966-mmc1.pdf]

Causes of excess non-lymphoma death in 58 000 patients with DLBCL diagnosed from 1997-2020 followed for up to 25 years

Supplemental Material

		Page
Supplemental Methods		2
Supplemental Table 1	Categorization of underlying cause of death using ICD-9/10 codes	10
Supplemental Table 2	Characteristics of the DLBCL cohort	13
Supplemental Table 3	Standardized mortality ratios and absolute excess mortality rates (per 10,000 person-years) for the DLBCL cohort by cause of death and time since diagnosis	15
Supplemental Figure 1	Exclusion criteria for the DLBCL cohort	17
Supplemental Figure 2	Mortality rate and cumulative risk for the DLBCL cohort by time since diagnosis, calendar period of diagnosis, and cause of death	18
Supplemental Figure 3	Standardized mortality ratios by cause of death for the DLBCL cohort during the first year after diagnosis, by age at diagnosis and calendar period of diagnosis	19
Supplemental Figure 4	Standardized mortality ratios by cause of death for the DLBCL cohort during years 1-4 after diagnosis, by age at diagnosis and calendar period of diagnosis	20
Supplemental Figure 5	Standardized mortality ratios by cause of death for the DLBCL cohort during years 5-11 after diagnosis, by age at diagnosis and calendar period of diagnosis	21
Supplemental Figure 6	Rate ratios and numbers of excess deaths by route to diagnosis (emergency vs non-emergency) for the 37,225 DLBCL patients diagnosed 2006-2018 for whom route to diagnosis was known, during the first year after diagnosis	22
Supplemental Figure 7	Rate ratios and numbers of excess deaths by route to diagnosis (emergency vs non-emergency) for the 37,225 DLBCL patients diagnosed 2006-2018 for whom route to diagnosis was known, during the first year after diagnosis, by age at diagnosis	23
Supplemental Figure 8	Excess non-lymphoma deaths and absolute excess mortality rates for the major causes of non-lymphoma death in the DLBCL cohort in patients diagnosed from 2011, by time since diagnosis	24
Supplemental Figure 9	Absolute excess mortality rates for the DLBCL cohort during the first year after diagnosis, by cause of death, gender and age at diagnosis	25
Supplemental Figure 10	Absolute excess mortality rates for the DLBCL cohort during the first year after diagnosis, by cause of death, age at diagnosis and calendar period of diagnosis	26
Supplemental Figure 11	Absolute excess mortality rates for the DLBCL cohort during years 1-14 after diagnosis, by cause of death, gender and age at diagnosis	27
Supplemental Figure 12	Cumulative risk of death for the DLBCL cohort, by time since diagnosis, cause of death and calendar period of diagnosis	28
Supplemental Figure 13	Rate ratios by recorded use of radiotherapy for the whole DLBCL cohort from 5 years after diagnosis	29
Supplemental Figure 14	Rate ratios by recorded use of radiotherapy for the DLBCL cohort from 1 year after diagnosis	30
Supplemental Figure 15	Rate ratios by recorded use of radiotherapy for the DLBCL cohort from 5 year after diagnosis	31

Supplemental Methods.

A. Data and study cohort

1. Datafiles

The central dataset of 349,747 patients was received from the National Cancer Registration and Analysis Service (NCRAS) and contained one record for every patient whose first registration of lymphoma in England was between 1st January 1997 to 31st December 2020 inclusive and on or after their 18th birthday. Patients who had previously been registered with a lymphoma in England were excluded. Patient records were anonymized and contained basic demographic information, including: gender; dates of birth, embarkation and death; and cause of death information, which was available from 1st January 1997 to 31st July 2023 inclusive.

Other datasets received from NCRAS included:

- The tumor registry, which had a row for each registered tumor (so potentially multiple rows per patient) and contained information about the tumor registration. The following were extracted regarding the index lymphoma: date of registration, Ann Arbor stage, morphology code (4-digit international classification of diseases (ICD)-O code), diagnostic classification code (mostly 4-digit ICD-9/10), histology description (text), National Health Service (NHS) region of diagnosis, Lower layer Super Output Area (LSOA) code at diagnosis (a small geographic unit in England used for statistical analysis, typically containing 1,000 to 3,000 residents or 400 to 1,200 households)[1], outward postcode at diagnosis, Charlson Comorbidity Index[2] (CCI) at diagnosis, and an indicator of whether the diagnosis was made using only the death certificate (DCO). The CCI accounted for all known comorbidities from 78 months to 6 months before the registration of the tumor.
- A dataset containing the quintile of the index of multiple deprivation[3], a measure of poverty, for each registered tumor for each patient. The deprivation quintile linked to the index lymphoma was extracted.
- Treatment registry, which had a row for each registered tumor and contained information regarding administered treatments. Treatment information linked to the index lymphoma was extracted.
- Systemic Anti-Cancer Therapy (SACT), which had a row for each cycle of systemic therapy administered (so potentially multiple rows per tumor per patient). Treatment information linked to the index lymphoma, the first recording of Ann Arbor stage for the index lymphoma, and ethnicity were extracted.
- Cancer waiting times, which contained information about the waiting times from referral to treatment. Treatment information linked to the index lymphoma was extracted.
- Route to diagnosis, which contained information about the pathway leading to diagnosis of each tumor. Both the route to diagnosis of the index lymphoma and an indicator of whether the diagnosis was DCO were extracted.

The variables extracted from these datasets were combined into a single dataset with one record per patient corresponding to information about the patient and their index lymphoma.

2. Data pre-processing

The following data pre-processing decisions were made:

- The date of diagnosis for the index tumor was taken to be the date of its registration.
- All patients who embarked (approx. 0.1% of all patients) during follow-up had a missing date of embarkation and were therefore removed from the dataset.
- As the date of birth only consisted of the month and year (i.e., mm/yyyy), the 15th was arbitrarily chosen as the day of birth for every patient (i.e., 15/mm/yyyy).
- Some of the diagnostic classification codes for the index tumors were in forms other than ICD-10. These were converted to ICD-10 using a conversion created by several members of our team.
- Information on deprivation quintile was not available for the patients diagnosed pre-1999 (6% of all patients). Therefore, as the deprivation quintile is completely determined by geography, a simple imputation was performed in these patients using the LSOA code and outward postcode at diagnosis. In particular, for every unique LSOA code present in the dataset, the earliest non-missing deprivation quintile that shared that LSOA code was extracted. This matching was used to estimate the missing deprivation data in patients diagnosed pre-1999. Following this, a very small number of patients (0.003% of all patients) still had a missing deprivation quintile, and so the same method was repeated using the outward postcodes instead of the LSOA codes, after which no deprivation information was missing.
- Each patient's deprivation quintile was taken to be their deprivation quintile at diagnosis of their index lymphoma and assumed not to change over follow-up.
- A patient was determined as receiving a particular treatment (e.g., radiotherapy) for their index lymphoma if they had a record of receiving that treatment linked to their index lymphoma either within 90 days before the date of diagnosis or within 1 year after the date of diagnosis. All treatments noted as being for recurrence, or any treatment with the same date as one noted as being for recurrence, were ignored.
- Where multiple datasets provided information on the same variable (e.g., Ann Arbor stage was available both in tumor registry and SACT), one of the datasets was chosen to take priority and other datasets were only used when values were missing in the priority dataset.

3. Underlying cause of death

In England, death certificates contain two parts: immediate cause of death and causes leading directly to death (part 1) and significant conditions/diseases that contributed to, but didn't directly cause, death (part 2). In the data received from NCRAS, causes are encoded using 4-digit ICD-9/10 codes. From the death certificate, one ICD-9/10 code representing the underlying cause of death was derived using the following rules, which were based on WHO guidelines[4].

If a single ICD code was supplied as the underlying cause, then this was accepted. When no underlying cause was given but some ICD codes were present in sections 1a-c or 2 of the death certificate, then the underlying cause was taken as the completed section with highest priority, where priority was given in the following descending order: 1c, 1b, 1a, 2 (i.e., information in section 2 was only considered if 1a-c were all empty). Where multiple ICD codes were given as the underlying cause, then, if cancer was mentioned, it took priority, with the following exception: if cancer is recorded on the lowest used line and there is mention of either hypertension, angina pectoris, ischemic heart disease or arteriosclerosis, on any of the lines above the cancer, then the cardiovascular condition is selected as the underlying cause. Cancer codes that specified the site of the cancer took priority over cancers of unspecified site and, if more than one primary cancer site

was specified, the first was taken. Where cancer codes were not mentioned, the most precise condition was prioritized (including ignoring post-procedural disorders).

In order to categories the ICD-9/10 codes of the underlying causes into clinically meaningful groups, a disease category classification table (supplemental Table 1) was developed by multiple oncologists in our team. Lymphoma (instead of DLBCL) was considered as an individual cause of death due to suspected inaccuracy of the sub-classification of lymphomas on the death certificate.

4. Study cohort

The classification of which patients had diffuse large B-cell lymphoma (DLBCL) as their index lymphoma was made by a clinical oncologist and a hematopathologist using the morphology ICD-O codes, the diagnostic classification ICD-10 codes, and the histological descriptions. A total of 74,444 of the index lymphomas were classified as DLBCL.

In order to represent the largest number of DLBCL patients and maximize statistical power, minimal exclusions (supplemental Figure 1) were performed when choosing the study cohort. The only exclusions were:

1. Patients whose index DLBCL could only be diagnosed through their death certificate and/or patients who had no follow-up.
2. Patients whose index DLBCL was diagnosed on or after their 80th birthday. This cut-of allows all patients to have at least 5 years of follow-up before they are censored at age 85 (see below).
3. Patients who died during follow-up but no information regarding their cause of death was available.

Similar studies commonly exclude patients with no recording of chemotherapy[5]. This was decided against in this study due to suspected large amounts of missing information, especially in earlier calendar periods (the recorded use of systemic therapy increased with era of diagnosis despite little change to first-line management of DLBCL in that time, see supplemental Table 2). Instead, it is reasonable to assume that almost all patients that survived at least one year since their DLBCL diagnosis must have received multi-agent chemotherapy due to the aggressive nature of the disease, the majority being anthracycline-based, i.e., CHOP/R-CHOP and related regimens.

Patients were considered to be at risk from the day after their diagnosis until the earliest of: date of death, date of 85th birthday, or the 31st December 2022. This date was chosen as the whole England population data was only available until the end of 2022, and the age cut-off was chosen as the whole England population data crudely groups all over-85s into the same age-group, which would have led to less precise age-standardization when comparing the cohort to the population (supplemental Methods B).

5. Malignant neoplasms of multiple/unspecified sites

Following a careful investigation, deaths from malignant neoplasm of multiple/unspecified neoplasms (MUN) were grouped with deaths from lymphoma. This decision was based on the following reasoning:

- Of the 503 patients whose underlying cause of death was MUN, only 269 (53%) had a registration of a cancer other than their index DLBCL anywhere in their cancer history. If

MUN deaths were due to non-lymphoma tumors, one would expect this number to be larger.

- Almost half (46%) of deaths from MUN occurred within the first year after DLBCL diagnosis, where the lymphoma-specific mortality rate is very high.

6. Whole England population cause of death data

Two datasets were provided by the Office of National Statistics regarding mortality in the wider English population (i.e., not just patients with lymphoma). The first contained the number of deaths by underlying cause (4-digit ICD-9/10 code) for each unique strata defined by: attained age (five-year categories until age 85 and then 85+ as one group); calendar year (from 1997-2022); gender; and deprivation quintile. The second dataset contained estimates of the whole English population size over the same strata. However, as this second dataset didn't include any deprivation information pre-2001, the deprivation-specific population structure of 2001 was applied to the pre-2001 years. Moreover, as this dataset also didn't also include any data at all for 2021-2022, the 2020 population estimates were used for these years.

B. Statistical methods

1. Flexible parametric models (Figure 1; supplemental Figure 2)

Flexible parametric models (FPMs) are parametric survival models that model the baseline hazard using a restricted cubic spline, with the number/position of internal knots specified by the user. See Royston and Parmar[6] for full details including estimation of confidence intervals.

To estimate the cause-specific hazard (Figure 1 left-panel) for deaths from lymphoma, $\hat{h}_{\mathrm{lym}}$, all patients in the cohort who were either alive at end of follow-up or had died from a non-lymphoma cause were censored, with only deaths from lymphoma considered as events. A FPM with two internal knots at years 1 and 5 after DLBCL diagnosis was fitted to model the hazard function $\hat{h}_{\mathrm{lym}}$. These knot positions were chosen for consistency with the time since diagnosis split points used throughout the paper and using only two internal knots was thought to be sufficient to capture the underlying shape of the baseline hazard. As a sensitivity check, the analysis was rerun using a variety of different knot positions and number of internal knots, and the results were more or less unchanged. The method to estimate the cause-specific hazard for deaths from all non-lymphoma causes, $\hat{h}_{\mathrm{no\_lym}}$, was analogous.

In order to estimate the cumulative risk (CR) of death from lymphoma adjusted for deaths from non-lymphoma as competing events (Figure 1 right-panel), the overall survival function was first estimated, which in turn required an estimate of the hazard function for deaths from all causes, $\hat{h}_{\mathrm{all\_cause}}$. This was obtained by splitting the follow-up into weekly intervals (rather than monthly for increased accuracy) and, for each interval, summing the respective hazard estimates for lymphoma and non-lymphoma causes using the models described in the previous paragraph, i.e.

$$\hat{h}_{\mathrm{all\_cause}}(t) = \hat{h}_{\mathrm{lym}}(t) + \hat{h}_{\mathrm{no\_lym}}(t), \qquad t = \frac{1}{52}, \frac{2}{52} \cdots$$

where t represents time since DLBCL diagnosis in years. The overall survival function at t years after diagnosis was estimated as

$$\hat{S}(t) = \exp\left(- \sum_{j=1/52}^{t} \frac{1}{52} \hat{h}_{\text{all\_cause}}(j) \right)$$

with $\hat{S}(0) \coloneqq 1$. The CR of mortality from lymphoma at time t was similarly estimated as

$$\widehat{\text{CR}}_{\text{lym}}(t) = \sum_{j=1/52}^{t} \frac{1}{52} \hat{h}_{\text{lym}}(j) \hat{S}(j - \frac{1}{52})$$

The method to estimate the CR of mortality from non-lymphoma causes adjusted for deaths from lymphoma was analogous.

For the analysis in supplemental Figure 2, the above was repeated separately for each calendar period of diagnosis.

2. Stratified rate ratios (Figure 4; supplemental Figures 6-7, 13-15)

To estimate the association between certain variables and cause-specific mortality, stratified rate ratios (RRs) were estimated using the conditional Poisson model. In supplemental Figure 6 and Figure 4, RRs were stratified by available variables that were thought to be potential confounders. Ann Arbor stage was not used for stratification due to high levels of missingness for all calendar periods (supplemental Table 2). Regarding the analyses for supplemental Figure 6 and Figure 4 in more detail:

- Supplemental Figure 6: Patients diagnosed before 2006 or after 2018 were excluded as the route to diagnosis was almost always missing during these years. Of the remaining 38,591 patients, 1366 (3.5%) were excluded as their route to diagnosis was missing, resulting in a sub-cohort of size 37,225. As a conservative sensitivity check for the effect of removing patients with missing values, the analysis was repeated by combining the 1366 patients with missing route to diagnosis with the patients with non-emergency presentation (i.e., emergency vs non-emergency/unknown), and the results were very similar to as shown in supplemental Figure 6.
- Figure 4: Patients diagnosed before 2006 were excluded as the CCI was missing in these patients. This resulted in a sub-cohort of size 45,025. In order to limit the number of statistical tests performed, only individual causes from the solid tumors or circulatory disease major groups with at least 20 deaths were included. We conducted three sensitivity analyses:
  A: Repeat the analysis for the whole cohort, i.e., also include patients diagnosed from 1997-2005 (supplemental Figure 13). In this case we didn't adjust for the CCI.
  B: Repeat the analysis where patients are determined to be at risk at 1 year after diagnosis rather than 5 years (supplemental Figure 14).
  C: Repeat the analysis with the cut-off time for recorded treatment to be included as 2 years instead of 1 (supplemental Figure 15).
  The main finding that recorded RT was associated with a reduction in mortality in cardiomyopathy/congestive heart failure was consistent across all sensitivity analyses.

3. Standardized mortality ratio, excess deaths and absolute excess mortality rate (Table 2; Figure 3; supplemental Table 3; supplemental Figures 3-5 & 8-11)

In order to calculate the standardized mortality ratio (SMR) for cause of death k, SMR_k, the follow-up of the DLBCL cohort was subdivided into distinct strata (labelled S_i) defined by: attained age (five-

year groups: 0-4, 5-9, …, 80-84), attained calendar year, gender at diagnosis and deprivation quintile. For each stratum S_i, let pyrs_i and O_i^k be the total number of person-years and the total number of deaths from underlying cause k in the stratum, respectively. Let $\mathrm{pop\_rate}_i^k$ be the death rate from cause k in stratum S_i in the wider English population. In each stratum S_i, the population rate was multiplied by the cohort person-years to estimate the expected number of deaths from cause k, i.e.

$$E_i^k = \mathrm{pyrs}_i * \mathrm{pop\_rate}_i^k$$

The SMR for cause k is defined as

$$\mathrm{SMR}_k = \frac{O^k}{E^k}$$

where $O^k = \sum_{i=1}^{N} O_i^k$ and $E^k = \sum_{i=1}^{N} E_i^k$ are the total observed and expected deaths from cause k across all strata, respectively. The SMR can be thought of as the relative difference in the mortality rate in the DLBCL cohort compared to a similar DLBCL-free cohort, with values of <1 indicating the DLBCL cohort dies at a lower rate and values of >1 at a higher (excess) rate. This interpretation relies on the standard assumption that the DLBCL cohort makes a negligible contribution to the death rate from cause k in the wider England population.

Exact confidence intervals for the SMR were calculated using the Poisson distribution, by assuming $O^k \sim \mathrm{Poisson}(E^k)$. Tests for linear trend (supplemental Figures 3-5) were performed using Poisson regression with the variable being tested encoded as continuous with labels from 1 to the number of categories. Likelihood ratio tests were used to estimate p-values.

The focus of the study was on the absolute size of excess mortality. To this end, we define two interrelated metrics. The number of excess deaths (EDs) for cause k is simply defined as

$$\mathrm{ED} = O^k - E^k$$

The absolute excess (mortality) rate (AER) for cause k (AER_k), per 10,000 person-years (pyrs), is the EDs scaled by the number of pyrs, i.e.

$$\mathrm{AER}_k := 10{,}000\frac{O^k - E^k}{\mathrm{pyrs}} = 10{,}000 * (\mathrm{SMR}_k - 1) * \frac{E^k}{\mathrm{pyrs}} \qquad (1)$$

where $\mathrm{pyrs} = \sum_{i=1}^{N} \mathrm{pyrs}_i$ is the total person-years in the DLBCL cohort, and the second equality follows from the definition of SMR_k. The AER can be thought of the additive difference in the death rate between the DLBCL cohort and a similar population without DLBCL, with values of <0 indicating the DLBCL cohort dies at a lower rate and values of >0 at a higher (excess) rate. The AER (and its confidence intervals) was estimated directly from the SMR (and its confidence intervals) using the second equality of (1).

Note that, where SMRs, EDs and AERs are given for individual causes that are only relevant for one gender, the calculations were performed in the subset of the cohort corresponding to that gender. This applied to the following individual causes: female breast cancer; cervix and uterine cancer; cancer of ovary or fallopian tubes; male breast cancer; prostate cancer; testicular cancer.

4. Observed and expected cumulative risk (Figure 2; supplemental Figure 12)

To aid the interpretability of mortality excesses, it is desirable to present estimates of observed and expected cumulative risk (CR). These two quantities are defined as follows: (a) the observed CR of death is the probability of death from cause k by time t as observed in the DLBCL cohort, accounting

for deaths from all other causes as competing events; (b) the expected CR is the probability of death from cause k by time t in the counterfactual scenario where the mortality rate from non-lymphoma causes was the same as in the wider England population, accounting for deaths from all other causes as competing events. The latter can equivalently be thought of as the expected CR in the DLBCL cohort in the scenario where the lymphoma-specific mortality was as was observed (factual), but there was no excess in non-lymphoma-specific mortality (counterfactual). This quantity is of interest as the main aim of this study is not to investigate excess mortality from lymphoma, which will obviously be large due to the nature of the cohort, but rather to investigate excess mortality from non-lymphoma causes. Let $\mathrm{CR}_k^{\mathrm{obs}}(t)$ and $\mathrm{CR}_k^{\mathrm{exp}}(t)$ be the quantities described by (a) and (b) respectively.

First regarding quantity (a), recall that we wish to approximate the following

$$\mathrm{CR}_k^{\mathrm{obs}}(t) = \int_0^t S^{\mathrm{obs}}(r) h_k^{\mathrm{obs}}(r)\, dr$$

where $S^{\mathrm{obs}}(t)$ is the observed overall survival function at time t and $h_k^{\mathrm{obs}}(t)$ is the observed cause-specific hazard function for cause k at time t. To this end, the cohort's follow-up was first split into monthly intervals. Let N_t, o_t and o_t^k respectively denote the number of patients at risk at the start the interval $\left[t - \frac{1}{12}, t\right)$, the number of deaths from all causes during the interval $\left[t - \frac{1}{12}, t\right)$, and the number of deaths from cause k during the interval $\left[t - \frac{1}{12}, t\right)$, where $t = \frac{1}{12}, \frac{2}{12}, \ldots$ represent monthly intervals encoded in years since diagnosis. We estimated $\mathrm{CR}_k^{\mathrm{obs}}(t)$ as

$$\widehat{\mathrm{CR}}_k^{\mathrm{obs}}(t) = \sum_{j=1/12}^{t} \hat{S}_{j-1/12}^{\mathrm{obs}} \frac{o_j^k}{N_j}$$

where $\hat{S}_t^{\mathrm{obs}}$ is an estimate of $S^{\mathrm{obs}}(t)$,

$$\hat{S}_t^{\mathrm{obs}} = \prod_{j=1/12}^{t} \left(1 - \frac{o_j}{N_j}\right)$$

and $\hat{S}_0^{\mathrm{obs}} := 1$. Note that $\widehat{\mathrm{CR}}_k^{\mathrm{obs}}(t)$ is similar to the Aalen-Johansen estimator[7] and $\hat{S}_t^{\mathrm{obs}}$ to the Kaplan-Meier estimator with the time-axis split at monthly intervals instead of at all uncensored failure times. Confidence intervals were estimated using the formulae in Coviello and Boggess[8].

Regarding the estimation of $\mathrm{CR}_k^{\mathrm{exp}}(t)$, let e_t and e_t^k respectively denote the expected number of deaths from all causes and the expected number of deaths from cause k, both during the month $\left[t - \frac{1}{12}, t\right)$. Recall that 'expected' refers to the scenario where the lymphoma-specific mortality is as observed in the cohort, and non-lymphoma-specific mortality is derived using the rates from the whole England population. To that end, e_t is estimated as

$$e_t = o_t^{\mathrm{lym}} + e_t^{\mathrm{no\_lym}}$$

where o_t^{lym} denotes the observed number of deaths from lymphoma in the cohort and $e_t^{\mathrm{no\_lym}}$ the expected number of deaths from non-lymphoma causes, both during the interval $\left[t - \frac{1}{12}, t\right)$. To estimate $e_t^{\mathrm{no\_lym}}$, and more generally e_t^k, we match the DLBCL cohort to the wider English population by attained age, attained calendar year, gender and deprivation quintile analogously to the SMR calculation (part 2), but this is now repeated for every month of follow-up. Finally, let

$$\widehat{\mathrm{CR}}_k^{\exp}(t) = \sum_{j=1/12}^{t} \hat{S}_{j-1/12}^{\exp} \frac{e_j^k}{N_j}$$

be our estimate for $\mathrm{CR}_k^{\exp}(t)$, where

$$\hat{S}_t^{\exp} = \prod_{j=1/12}^{t} \left(1 - \frac{e_j}{N_j}\right)$$

is our estimate for $S^{\exp}(t)$, the expected survival function. Despite the dependence of $\widehat{\mathrm{CR}}_k^{\exp}(t)$ on the observed deaths through o_t^{lym}, confidence intervals were not plotted for ease of visualization.

For the analysis in supplemental Figure 12, the above was repeated separately for each calendar period of diagnosis.

References

1. Office for National Statistics, Statistical geographies, https://www.ons.gov.uk/methodology/geography/ukgeographies/statisticalgeographies, Accessed 28 April 2025.
2. NDRS, Comorbidities and cancer, https://digital.nhs.uk/ndrs/our-work/ncras-work-programme/comorbidities-and-cancer, Accessed 28 April 2025.
3. GOV.UK, English indices of deprivation 2019, https://www.gov.uk/government/statistics/english-indices-of-deprivation-2019, Accessed 28 April 2025.
4. WHO, International statistical classification of diseases and related health problems, ICD-10 Volume 2, https://www.who.int/publications/m/item/international-statistical-classification-of-diseases-and-related-health-problems---volume-2, Accessed 28 April 2025.
5. Bei L-Y, Shang C-Y, Wu J-Z, et al. Cause-specific mortality in a population-level cohort of diffuse large B-cell lymphoma following chemotherapy in the early 21st century. *Ann Hematol*. 2024;103(5):1675–1685.
6. Royston P, Parmar MKB. Flexible parametric proportional-hazards and proportional-odds models for censored survival data, with application to prognostic modelling and estimation of treatment effects. *Statistics in Medicine*. 2002;21(15):2175–2197.
7. Aalen OO, Johansen S. An Empirical Transition Matrix for Non-Homogeneous Markov Chains Based on Censored Observations. *Scandinavian Journal of Statistics*. 1978;5(3):141–150.
8. Coviello V, Boggess M. Cumulative Incidence Estimation in the Presence of Competing Risks. *The Stata Journal*. 2004;4(2):103–112.

Supplemental Table 1. Categorization of underlying cause of death using ICD-9/10 codes. Each ICD code is assigned both an individual and a major cause of death group.

ICD-9	ICD-10	Individual cause of death group	Major cause of death group
200, 201, 202.0-202.2, 202.8	C81-C86, C88.0	Lymphoma (excl multiple and unspecified neoplasms)	Lymphoma
195-199	C76-C80, C97	Malignant neoplasm of unspecified sites or multiple sites	Lymphoma
	U07.1-U07.9, U08-U10	COVID-19	All infections
460-466, 473, 474.0, 475, 480-487, 494, 507, 510, 513	J00-J22, J32, J34.0, J35.0, J36, J39.0-J39.1, J47, J69, J85-J86	Pneumonia and other respiratory infections	All infections
001-139	A00-A99, B00-B99, U04.9	Other infections	All infections
204.0, 204.2, 205.0, 205.2, 205.3, 206.0, 206.2, 207.0, 207.2, 208.0, 208.2	C91.0, C91.3, C91.5, C91.6, C91.8, C92.0, C92.3-C92.6, C92.8, C93.0, C94.0, C94.2-C94.5, C95.0	Acute leukemia	All hematological (excl lymphoma)
204.1, 205.1, 206.1, 207.1, 208.1	C91.1, C91.4, C92.1, C92.2, C93.1-C93.3, C94.1, C95.1	Chronic leukemia	All hematological (excl lymphoma)
204.8, 204.9, 205.8, 205.9, 206.8, 206.9, 207.8, 208.8, 208.9	C91.7, C91.9, C92.7, C92.9, C93.7, C93.9, C94.6, C94.7, C95.7, C95.9	Other leukemia	All hematological (excl lymphoma)
159.1, 202.3-202.6, 202.9, 203.8, 238.4-238.7	C26.1, C88.1-C88.9, C96, D45-D47	MDS and other hematological neoplasms	All hematological (excl lymphoma)
203.0, 203.1	C90	Myeloma	All hematological (excl lymphoma)
288.0	D70	Agranulocytosis (neutropenia)	All hematological (excl lymphoma)
279-287, 288.1-288.9, 289	D50-D69, D71-D89	Other diseases of blood and blood forming organs	All hematological (excl lymphoma)
150	C15	Esophagus cancer	All gastrointestinal cancers; All solid tumors
151	C16	Stomach cancer	All gastrointestinal cancers; All solid tumors
153, 154.0, 154.1, 159.0	C18-C20	Colorectal cancer	All gastrointestinal cancers; All solid tumors
157	C25	Pancreas cancer	All gastrointestinal cancers; All solid tumors
152, 154.2, 154.3, 154.8, 155, 156, 158, 159.8, 159.9	C17, C21-C23, C24, C26.0, C26.8-C26.9, C45.1, C48	Other gastrointestinal cancer	All gastrointestinal cancers; All solid tumors
140, 141-149, 160, 161	C00-C14, C30-C32	Head and neck cancers	All solid tumors
162.2-162.5, 162.8-162.9	C34	Lung cancer	All solid tumors
162.0, 163-165	C33, C37-C39, C45.0, C45.2	Other respiratory and intrathoracic cancer	All solid tumors

170, 171, 176	C40, C41, C45.7, C45.9, C46, C47, C49	Cancer of soft tissue, bones and joints	All solid tumors
172	C43	Melanoma of skin	All solid tumors
173	C44	Other malignant neoplasms of skin	All solid tumors
174-175	C50	Breast cancer	All solid tumors
179, 180, 182	C53, C54, C55	Cervix and uterine cancer	All solid tumors
181, 183	C56-C58	Cancer of ovary or fallopian tubes	All solid tumors
185	C61	Prostate cancer	All solid tumors
186	C62	Testicular cancer	All solid tumors
189.0	C64	Kidney cancer	All solid tumors
188, 189.1-189.9	C65-C68	Bladder (and other urothelial) cancer	All solid tumors
184, 187	C51, C52, C60, C63	Other genitourinary cancers	All solid tumors
190-192, 224, 225, 227.3, 227.4, 234.0, 237.0, 237.1, 237.5, 237.6, 237.9, 239.6	C69-C72, D09.2, D31-D33, D35.2-4, D42-D43, D44.3-5	Tumors of central nervous system and eye	All solid tumors
193	C73	Thyroid cancer	All solid tumors
194	C74-C75	Other endocrine gland cancer	All solid tumors
210-223, 226-227.1, 227.5-233.9, 234.8-236.9, 237.2-237.4, 237.7-237.8, 238.0-238.3, 238.8-238.9, 239.0-239.5, 239.7-239.9	D00-D09.1, D09.3-D30.9, D34-D35.1, D35.5-D41.9, D44.0-2, D44.6-D44.9, D48	In-situ, benign or uncertain (excl hem and CNS/eye)	All solid tumors
410-414, 429.2	I20-I25	Ischemic heart disease	All cardiac; All circulatory
391.2, 398.0, 422, 425, 428, 429.0, 429.1, 429.3	I01.2, I09.0, I11.0, I13.0, I13.2, I40-I43, I50, I51.4-5, I51.7	Cardiomyopathy and congestive heart failure	All cardiac; All circulatory
391.1, 394-397, 424	I01.1, I05-I08, I09.1, I34-I39	Valvular heart disease	All cardiac; All circulatory
426, 427	I44-I49	Arrhythmias	All cardiac; All circulatory
391.0, 391.8-391.9, 392.0,392.9, 393, 398.9, 402, 404, 416.1-416.9, 420, 421, 429.4-429.9, 423	I01.0, I01.8-9, I02, I09.2, I09.8-9, I11.9, I13.1, I13.9, I27.1-9, I30-I33, I51.0-3, I51.6, I51.8, I51.9, I52	Other heart	All cardiac; All circulatory
430-438	I60-I69	Cerebrovascular	All circulatory
415, 444, 451-453	I26, I74,I80-I82	Thromboembolic	All circulatory
390, 401, 403, 405, 416.0, 417, 440-443, 446-448, 454-459	I00, I10, I12, I15, I27.0, I28, I70-I73, I77-I79, I83-I89, I95-I99	Aortic aneurysm, peripheral vascular and other circulatory	All circulatory
240-278	E00-E90	Endocrine and metabolic diseases	All causes other than hematological, solid tumor, circulatory and infection

290, 331, 797	F00-F03, G30-G31, R54	Dementia	All causes other than hematological, solid tumor, circulatory and infection
291-330, 332-359	F04-F99, G00-G26, G32-G99	Other neurological diseases	All causes other than hematological, solid tumor, circulatory and infection
490-493, 496	J40-J46	COPD and asthma	All causes other than hematological, solid tumor, circulatory and infection
470-472, 474.1-474.9, 476-479, 495, 497-506, 508-509, 511-512, 514-519	J30-J31, J33, J34.1-J34.9, J35.1-J35.9, J37-J38, J39.2-J39.9, J48-J68, J70-J84, J87-J99	Other respiratory diseases	All causes other than hematological, solid tumor, circulatory and infection
530-579	K20-K67, K70-K77, K80-K93	Digestive system disease	All causes other than hematological, solid tumor, circulatory and infection
680-709	L00-L99	Diseases of skin and subcutaneous tissue	All causes other than hematological, solid tumor, circulatory and infection
710-739	M00-M79, M81-M99	Musculoskeletal or soft tissue disease	All causes other than hematological, solid tumor, circulatory and infection
580-629	N00-N39	Kidney and urological disease	All causes other than hematological, solid tumor, circulatory and infection
360-389, 520-529, 630-679, 740-759,760-779	K00-K14, N40-N99, H00-H99, O00-O99, P00-P99, Q00-Q99, U07.0	Other specified diseases	All causes other than hematological, solid tumor, circulatory and infection
780-796, 798-799	R00-R53, R55-R99, U50, U99	Ill-defined diseases	All causes other than hematological, solid tumor, circulatory and infection
E950-E959	X60-X84	Suicide	All causes other than hematological, solid tumor, circulatory and infection
800-999, E800-E949, E960-E999, V01-V91	M80, S00-S99, T00-T99, V00-V99, W00-W99, X00-X59, X85-X99, Y00-Y99	External causes (excl suicide)	All causes other than hematological, solid tumor, circulatory and infection

Abbreviations: ICD, international classification of diseases; MDS, myelodysplastic syndrome; CNS, central nervous system; COPD, chronic obstructive pulmonary disease

Supplemental Table 2. Characteristics of the DLBCL cohort.

Characteristic		Calendar period of diagnosis				
		1997-2005 N (%)	2006-2010 N (%)	2011-2015 N (%)	2016-2020 N (%)	**All patients N (%)**
**Age at diagnosis (years)**	18-44	1,718 (13.0)	1,450 (11.3)	1,558 (9.8)	1,502 (9.2)	6,228 (10.7)
	45-59	3,213 (24.3)	2,871 (22.5)	3,346 (21.0)	3,407 (20.8)	12,837 (22.0)
	60-69	3,715 (28.2)	3,804 (29.8)	4,976 (31.3)	4,670 (28.6)	17,165 (29.5)
	70-79	4,550 (34.5)	4,657 (36.4)	6,028 (37.9)	6,756 (41.4)	21,991 (37.8)
**Gender**	Male	7,286 (55.2)	7,137 (55.8)	8,994 (56.5)	9,413 (57.6)	32,830 (56.4)
	Female	5,910 (44.8)	5,645 (44.2)	6,914 (43.5)	6,922 (42.4)	25,391 (43.6)
**Ethnicity**	White	10,815 (95.1)	11,556 (92.8)	14,093 (91.3)	14,097 (90.2)	50,561 (92.1)
	Mixed	37 (0.3)	65 (0.5)	71 (0.5)	105 (0.7)	278 (0.5)
	South Asian	276 (2.4)	443 (3.6)	685 (4.4)	764 (4.9)	2,168 (3.9)
	Black	133 (1.2)	230 (1.8)	311 (2.0)	300 (1.9)	974 (1.8)
	Chinese	25 (0.2)	33 (0.3)	45 (0.3)	64 (0.4)	167 (0.3)
	Other	87 (0.8)	124 (1.0)	226 (1.5)	304 (1.9)	741 (1.4)
	Unknown	1,823 (NA)	331 (NA)	477 (NA)	701 (NA)	3,332 (NA)
**Deprivation quintile**	<20% (least deprived)	2,873 (21.8)	2,714 (21.2)	3,453 (21.7)	3,409 (20.9)	12,449 (21.4)
	20-39%	2,914 (22.1)	2,819 (22.1)	3,446 (21.6)	3,528 (21.6)	12,707 (21.8)
	40-59%	2,674 (20.2)	2,607 (20.4)	3,242 (20.4)	3,379 (20.7)	11,902 (20.4)
	60-79%	2,428 (18.4)	2,412 (18.9)	3,082 (19.4)	3,109 (19.0)	11,031 (19.0)
	80+% (most deprived)	2,307 (17.5)	2,230 (17.4)	2,685 (16.9)	2,910 (17.8)	10,132 (17.4)
**NHS region**	South West	1,656 (12.5)	1,388 (10.9)	1,807 (11.4)	2,010 (12.3)	6,861 (11.8)
	North West	1,411 (10.7)	1,481 (11.6)	2,033 (12.8)	2,038 (12.5)	6,963 (12.0)
	East of England	1,200 (9.1)	1,601 (12.5)	2,013 (12.6)	1,926 (11.8)	6,740 (11.6)
	Midlands	2,814 (21.3)	2,686 (21.0)	3,066 (19.3)	3,182 (19.5)	11,748 (20.2)
	South East	2,397 (18.2)	2,017 (15.8)	2,596 (16.3)	2,630 (16.1)	9,640 (16.5)
	London	895 (6.8)	1,282 (10.0)	1,802 (11.3)	1,924 (11.8)	5,903 (10.1)
	North East and Yorkshire	2,823 (21.4)	2,327 (18.2)	2,591 (16.3)	2,625 (16.0)	10,366 (17.8)
**Charlson Comorbidity Index**	0	0 (NA)	10,225 (80.0)	11,914 (74.9)	11,608 (71.1)	33,747 (75.0)
	1	0 (NA)	1,319 (10.3)	1,997 (12.6)	2,183 (13.4)	5,499 (12.2)
	2+	0 (NA)	1,238 (9.7)	1,992 (12.5)	2,527 (15.5)	5,757 (12.8)
	Unknown	13,196 (NA)	0 (NA)	5 (NA)	17 (NA)	13,218 (NA)
**Previous cancer**	None recorded	11,887 (90.1)	11,147 (87.2)	13,408 (84.3)	13,390 (82.0)	49,832 (85.6)
	Previous cancer recorded	1,309 (9.9)	1,635 (12.8)	2,500 (15.7)	2,945 (18.0)	8,389 (14.4)
**Route to diagnosis**	TWW	0 (NA)	3,023 (24.6)	4,642 (30.2)	3,209 (33.5)	10,874 (29.2)
	GP referral	0 (NA)	3,493 (28.5)	3,857 (25.1)	2,044 (21.3)	9,394 (25.2)
	Other outpatient	0 (NA)	1,391 (11.3)	1,497 (9.7)	970 (10.1)	3,858 (10.4)
	Emergency presentation	0 (NA)	3,996 (32.6)	5,108 (33.2)	3,149 (32.9)	12,253 (32.9)
	Inpatient elective	0 (NA)	362 (3.0)	276 (1.8)	209 (2.2)	847 (2.3)
	Unknown	13,196 (NA)	517 (NA)	528 (NA)	6,754 (NA)	20,995 (NA)
**Ann Arbor stage**	0-2	1,214 (50.6)	1,379 (49.0)	4,501 (40.4)	4,550 (30.8)	11,644 (37.4)
	3-4	1,184 (49.4)	1,437 (51.0)	6,627 (59.6)	10,208 (69.2)	19,456 (62.6)
	Unstageable/unknown	10,798 (NA)	9,966 (NA)	4,780 (NA)	1,577 (NA)	27,121 (NA)
**Systemic therapy**	Systemic therapy recorded	8,430 (63.9)	10,101 (79.0)	13,956 (87.7)	14,641 (89.6)	47,128 (80.9)
	None recorded	4,766 (36.1)	2,681 (21.0)	1,952 (12.3)	1,694 (10.4)	11,093 (19.1)
**Radiotherapy**	Radiotherapy recorded	2,906 (22.0)	2,688 (21.0)	4,139 (26.0)	4,913 (30.1)	14,646 (25.2)
	None recorded	10,290 (78.0)	10,094 (79.0)	11,769 (74.0)	11,422 (69.9)	43,575 (74.8)

Characteristic		Calendar period of diagnosis 1997-2005 N (%)	2006-2010 N (%)	2011-2015 N (%)	2016-2020 N (%)	**All patients N (%)**
**Combined therapy**	Both therapies recorded	2,192 (16.6)	2,336 (18.3)	3,880 (24.4)	4,739 (29.0)	13,147 (22.6)
	Only Systemic therapy recorded	6,238 (47.3)	7,765 (60.7)	10,076 (63.3)	9,902 (60.6)	33,981 (58.3
	Only radiotherapy recorded	714 (5.4)	352 (2.8)	259 (1.6)	174 (1.1)	1,499 (2.6)
	None recorded	4,052 (30.7)	2,329 (18.2)	1,693 (10.7)	1,520 (9.3)	9,594 (16.5)
**Duration of follow-up (years)**	<1	3,895 (29.5)	3,286 (25.7)	3,711 (23.3)	3,789 (23.2)	14,681 (25.2)
	1-4	2,499 (18.9)	2,122 (16.6)	2,614 (16.4)	8,498 (52.0)	15,733 (27.0)
	5-9	1,775 (13.5)	1,941 (15.2)	7,010 (44.1)	4,048 (24.8)	14,774 (25.4)
	10-25	5,027 (38.1)	5,433 (42.5)	2,573 (16.2)	0 (0.0)	13,033 (22.4)
**Vital status at end of follow-up**	Alive	4,018 (30.4)	5,334 (41.7)	8,117 (51.0)	10,156 (62.2)	27,625 (47.4)
	Lymphoma	6,004 (45.5)	4,777 (37.4)	5,372 (33.8)	4,600 (28.2)	20,753 (35.6)
	All non-lymphoma causes	3,174 (24.1)	2,671 (20.9)	2,419 (15.2)	1,579 (9.7)	9,843 (17.0)
	Infection	353 (2.7)	321 (2.5)	330 (2.1)	413 (2.5)	1,417 (2.4)
	Hematological excl lymphoma	167 (1.3)	217 (1.7)	218 (1.4)	134 (0.8)	736 (1.3)
	Solid tumors	851 (6.4)	700 (5.5)	617 (3.9)	344 (2.1)	2,512 (4.3)
	Circulatory	1,044 (7.9)	689 (5.4)	541 (3.4)	281 (1.7)	2,555 (4.4)
	All other non-lymphoma causes	759 (5.8)	744 (5.8)	713 (4.5)	407 (2.5)	2,623 (4.5)
**Total**		13,196 (100.0)	12,782 (100.0)	15,908 (100.0)	16,335 (100.0)	

*Data on Charlson Comorbidity Index always unavailable in patients diagnosed before 2006

†Data on route to diagnosis was unavailable in patients diagnosed before 2006 or after 2018

Unknown values do not contribute towards column percentages

Abbreviations: N, number of DLBCL patients; NHS, National Health Service; NA, not applicable; TWW, two-week wait; GP, general practitioner

Supplemental Table 3. Standardized mortality ratios and absolute excess mortality rates (per 10,000 person-years) for the DLBCL cohort by cause of death and time since diagnosis. SMRs and AERs were calculated by comparing to all England rates accounting for the same calendar period, attained age, gender and deprivation quintile.

Time since diagnosis:	<1 year			1-4 years			5-9 years			10-25 years			Overall		
Cause of death	Obs	SMR (95% CI)	AER (95% CI)	Obs	SMR (95% CI)	AER (95% CI)	Obs	SMR (95% CI)	AER (95% CI)	Obs	SMR (95% CI)	AER (95% CI)	Obs	SMR (95% CI)	AER (95% CI)
All causes	14,681	19.5 (19.2, 19.9)	2861 (2812, 2910)	9,468	3.81 (3.73, 3.89)	499 (486, 513)	4,072	2.06 (2.00, 2.12)	212 (199, 224)	2,375	1.94 (1.87, 2.02)	186 (171, 202)	30,596	4.75 (4.70, 4.81)	691 (682, 701)
Lymphoma (excl multiple and unspecified neoplasms)	11,931	NA	NA	6,419	NA	NA	1,387	NA	NA	559	NA	NA	20,296	NA	NA
Malignant neoplasm of unspecified sites or multiple sites	230	11.6 (10.1, 13.2)	43 (37, 50)	102	1.71 (1.39, 2.08)	3.0 (1.7, 4.6)	73	1.68 (1.32, 2.11)	3.0 (1.4, 4.9)	52	2.11 (1.57, 2.76)	4.4 (2.3, 7.0)	457	3.09 (2.82, 3.39)	8.8 (7.7, 10.1)
Lymphoma	12,161	NA	NA	6,521	NA	NA	1,460	NA	NA	611	NA	NA	20,753	NA	NA
All causes excl lymphoma	2,520	3.49 (3.35, 3.63)	369 (349, 390)	2,947	1.23 (1.19, 1.27)	39 (32, 47)	2,612	1.37 (1.31, 1.42)	71 (61, 81)	1,764	1.49 (1.42, 1.56)	94 (81, 107)	9,843	1.58 (1.55, 1.61)	104 (98, 109)
COVID-19*	127	16.0 (13.4, 19.1)	24 (20, 29)	112	2.37 (1.95, 2.85)	4.6 (3.2, 6.2)	83	1.61 (1.28, 2.00)	3.2 (1.5, 5.2)	84	1.73 (1.38, 2.14)	5.7 (3.0, 8.9)	406	2.61 (2.36, 2.88)	7.2 (6.1, 8.4)
Pneumonia and other respiratory infections	144	4.52 (3.82, 5.33)	23 (18, 28)	144	1.31 (1.11, 1.54)	2.4 (0.8, 4.3)	130	1.45 (1.21, 1.72)	4.1 (1.9, 6.5)	102	1.94 (1.58, 2.36)	8.0 (5.0, 11.5)	520	1.83 (1.68, 2.00)	6.8 (5.5, 8.1)
Other infections	297	42.8 (38.1, 48.0)	60 (53, 67)	102	4.25 (3.46, 5.16)	5.6 (4.2, 7.1)	67	3.44 (2.67, 4.37)	4.8 (3.3, 6.6)	25	2.13 (1.38, 3.15)	2.1 (0.7, 4.1)	491	7.90 (7.22, 8.63)	12 (11, 14)
All infections	568	12.2 (11.2, 13.2)	107 (98, 117)	358	1.98 (1.78, 2.19)	13 (10, 15)	280	1.74 (1.54, 1.96)	12 (9, 16)	211	1.87 (1.63, 2.14)	16 (11, 21)	1,417	2.83 (2.68, 2.98)	26 (24, 28)
Acute leukemia	44	8.65 (6.29, 11.62)	8.0 (5.5, 11.1)	111	6.81 (5.61, 8.21)	6.8 (5.4, 8.4)	55	4.31 (3.25, 5.61)	4.3 (2.9, 6.0)	32	4.02 (2.75, 5.67)	3.9 (2.2, 6.0)	242	5.75 (5.05, 6.52)	5.7 (4.9, 6.6)
Chronic leukemia	34	15.2 (10.6, 21.3)	6.5 (4.4, 9.3)	31	4.15 (2.82, 5.90)	1.7 (1.0, 2.6)	15	2.49 (1.39, 4.11)	0.9 (0.2, 1.9)	7	1.89 (0.76, 3.90)	0.5 (-0.1, 1.7)	87	4.48 (3.59, 5.53)	1.9 (1.4, 2.5)
Other leukemia	5	11.3 (3.7, 26.5)	0.9 (0.2, 2.3)	4	2.99 (0.81, 7.66)	0.2 (-0.0, 0.6)	3	3.14 (0.65, 9.19)	0.2 (-0.0, 0.8)	0	0.00 (0.00, 7.89)	-0.1 (-0.1, 0.5)	12	3.75 (1.94, 6.55)	0.2 (0.1, 0.5)
MDS and other hematological neoplasms	109	50.6 (41.5, 61.0)	22 (18, 27)	64	7.86 (6.05, 10.04)	4.0 (2.9, 5.3)	34	4.86 (3.36, 6.79)	2.7 (1.7, 4.1)	15	3.32 (1.86, 5.48)	1.7 (0.6, 3.3)	222	10.2 (8.9, 11.6)	5.7 (4.9, 6.6)
Myeloma	20	3.88 (2.37, 5.99)	3.0 (1.4, 5.3)	24	1.43 (0.92, 2.13)	0.5 (-0.1, 1.4)	15	1.13 (0.63, 1.87)	0.2 (-0.5, 1.2)	8	0.97 (0.42, 1.90)	0.0 (-0.8, 1.2)	67	1.54 (1.20, 1.96)	0.7 (0.2, 1.2)
Agranulocytosis (neutropenia)	10	45.9 (22.0, 84.3)	2.0 (0.9, 3.7)	3	4.49 (0.93, 13.14)	0.2 (-0.0, 0.6)	5	9.88 (3.21, 23.05)	0.4 (0.1, 1.1)	1	3.50 (0.09, 19.50)	0.1 (-0.0, 0.8)	19	11.3 (6.8, 17.7)	0.5 (0.3, 0.8)
Other diseases of blood and blood forming organs	40	31.2 (22.3, 42.5)	8.0 (5.6, 10.9)	24	5.98 (3.83, 8.90)	1.4 (0.8, 2.3)	17	5.42 (3.16, 8.67)	1.4 (0.7, 2.4)	6	3.03 (1.11, 6.59)	0.6 (0.0, 1.8)	87	8.35 (6.69, 10.30)	2.2 (1.7, 2.8)
All hematological (excl lymphoma)	262	15.8 (14.0, 17.9)	50 (44, 57)	261	4.78 (4.21, 5.39)	15 (13, 17)	144	3.30 (2.78, 3.88)	10 (8, 13)	69	2.54 (1.97, 3.21)	6.8 (4.3, 9.7)	736	5.18 (4.81, 5.57)	17 (16, 19)
Esophagus cancer	6	0.41 (0.15, 0.89)	-1.8 (-2.6, -0.3)	46	1.02 (0.74, 1.36)	0.0 (-0.8, 1.1)	46	1.35 (0.99, 1.80)	1.2 (-0.0, 2.8)	37	1.75 (1.24, 2.42)	2.6 (0.8, 4.8)	135	1.17 (0.98, 1.39)	0.6 (-0.1, 1.3)
Stomach cancer	41	4.75 (3.41, 6.45)	6.6 (4.3, 9.6)	20	0.79 (0.48, 1.22)	-0.4 (-0.9, 0.4)	23	1.26 (0.80, 1.89)	0.5 (-0.4, 1.6)	9	0.87 (0.40, 1.66)	-0.2 (-1.0, 1.1)	93	1.49 (1.20, 1.82)	0.9 (0.4, 1.5)
Colorectal cancer	33	1.22 (0.84, 1.72)	1.2 (-0.9, 4.0)	87	1.03 (0.83, 1.27)	0.2 (-1.1, 1.6)	80	1.23 (0.98, 1.54)	1.5 (-0.1, 3.5)	57	1.45 (1.10, 1.87)	2.8 (0.6, 5.6)	257	1.19 (1.05, 1.35)	1.2 (0.3, 2.1)
Pancreas cancer	26	1.63 (1.07, 2.39)	2.1 (0.2, 4.6)	36	0.71 (0.50, 0.98)	-1.1 (-1.8, -0.1)	47	1.18 (0.87, 1.57)	0.7 (-0.5, 2.3)	53	2.09 (1.57, 2.74)	4.5 (2.3, 7.1)	162	1.23 (1.05, 1.43)	0.9 (0.2, 1.6)
Other gastrointestinal cancer	37	2.31 (1.63, 3.19)	4.3 (2.1, 7.2)	67	1.25 (0.97, 1.58)	0.9 (-0.1, 2.2)	58	1.32 (1.01, 1.71)	1.4 (0.0, 3.1)	43	1.51 (1.09, 2.03)	2.3 (0.4, 4.8)	205	1.44 (1.25, 1.65)	1.8 (1.0, 2.7)
All gastrointestinal cancers	143	1.74 (1.46, 2.05)	12 (8, 18)	256	0.99 (0.87, 1.11)	-0.3 (-2.4, 2.1)	254	1.26 (1.11, 1.43)	5.4 (2.3, 8.7)	199	1.60 (1.38, 1.83)	12 (8, 17)	852	1.28 (1.19, 1.37)	5.3 (3.7, 7.0)
Head and neck cancers	8	1.25 (0.54, 2.46)	0.3 (-0.6, 1.9)	27	1.37 (0.91, 2.00)	0.5 (-0.1, 1.4)	29	1.95 (1.31, 2.80)	1.4 (0.5, 2.7)	20	2.04 (1.24, 3.15)	1.6 (0.4, 3.4)	84	1.66 (1.32, 2.05)	0.9 (0.5, 1.5)
Lung cancer	58	0.85 (0.65, 1.11)	-2.0 (-4.9, 1.5)	212	1.05 (0.91, 1.20)	0.7 (-1.3, 2.9)	233	1.55 (1.36, 1.76)	8.3 (5.4, 11.6)	152	1.69 (1.43, 1.98)	10.0 (6.2, 14.2)	655	1.28 (1.19, 1.38)	4.1 (2.7, 5.6)
Other respiratory and intrathoracic cancer	6	4.41 (1.62, 9.60)	0.9 (0.2, 2.4)	2	0.51 (0.06, 1.85)	-0.1 (-0.3, 0.2)	3	1.08 (0.22, 3.15)	0.0 (-0.2, 0.6)	1	0.64 (0.02, 3.54)	-0.1 (-0.2, 0.6)	12	1.25 (0.64, 2.18)	0.1 (-0.1, 0.3)
Cancer of soft tissue, bones and joints	12	2.04 (1.05, 3.56)	1.2 (0.1, 3.1)	24	1.27 (0.81, 1.88)	0.4 (-0.3, 1.2)	18	1.21 (0.72, 1.91)	0.3 (-0.4, 1.4)	14	1.50 (0.82, 2.52)	0.8 (-0.3, 2.3)	68	1.39 (1.08, 1.76)	0.5 (0.1, 1.1)
Melanoma of skin	2	0.54 (0.07, 1.96)	-0.3 (-0.7, 0.7)	26	2.21 (1.45, 3.24)	1.0 (0.4, 1.9)	22	2.39 (1.50, 3.62)	1.3 (0.5, 2.4)	11	1.89 (0.94, 3.38)	0.8 (-0.0, 2.2)	61	2.00 (1.53, 2.57)	0.9 (0.5, 1.4)
Other malignant neoplasms of skin	2	2.33 (0.28, 8.42)	0.2 (-0.1, 1.3)	5	1.55 (0.50, 3.63)	0.1 (-0.1, 0.6)	11	3.92 (1.95, 7.01)	0.8 (0.3, 1.7)	6	3.16 (1.16, 6.88)	0.7 (0.0, 1.8)	24	2.73 (1.75, 4.07)	0.4 (0.2, 0.8)
Male breast cancer†	1	6.03 (0.15, 33.59)	0.3 (-0.0, 2.0)	1	1.99 (0.05, 11.07)	0.1 (-0.1, 0.6)	0	0.00 (0.00, 9.93)	-0.1 (-0.1, 0.6)	1	4.31 (0.11, 24.03)	0.2 (-0.1, 1.6)	3	2.36 (0.49, 6.89)	0.1 (-0.0, 0.4)
Female breast cancer†	17	1.14 (0.66, 1.82)	1.0 (-2.4, 5.8)	25	0.55 (0.35, 0.81)	-3.3 (-4.8, -1.4)	38	1.10 (0.78, 1.51)	0.8 (-1.7, 3.9)	32	1.54 (1.05, 2.17)	4.0 (0.4, 8.7)	112	0.96 (0.79, 1.16)	-0.3 (-1.5, 1.2)
Cervix and uterine cancer†	8	1.90 (0.82, 3.74)	1.8 (-0.4, 5.4)	10	0.74 (0.36, 1.37)	-0.6 (-1.4, 0.8)	13	1.22 (0.65, 2.08)	0.5 (-0.8, 2.6)	7	1.03 (0.42, 2.13)	0.1 (-1.4, 2.7)	38	1.08 (0.77, 1.48)	0.2 (-0.5, 1.1)
Cancer of ovary or fallopian tubes†	8	1.16 (0.50, 2.29)	0.5 (-1.6, 4.2)	23	1.10 (0.70, 1.65)	0.3 (-1.0, 2.2)	14	0.89 (0.49, 1.49)	-0.4 (-1.8, 1.7)	11	1.15 (0.57, 2.05)	0.5 (-1.5, 3.6)	56	1.05 (0.80, 1.37)	0.2 (-0.7, 1.2)
Prostate cancer†	13	0.68 (0.36, 1.17)	-2.2 (-4.4, 1.2)	50	0.78 (0.58, 1.03)	-1.8 (-3.5, 0.2)	59	1.16 (0.88, 1.49)	1.5 (-1.1, 4.6)	31	1.00 (0.68, 1.42)	0.0 (-2.9, 3.8)	153	0.93 (0.79, 1.09)	-0.6 (-1.8, 0.7)
Testicular cancer†	1	13.6 (0.3, 75.9)	0.3 (-0.0, 2.0)	1	4.58 (0.12, 25.50)	0.1 (-0.0, 0.7)	1	6.49 (0.16, 36.13)	0.2 (-0.0, 1.0)	0	0.00 (0.00, 36.48)	0.0 (-0.0, 1.0)	3	5.48 (1.13, 16.02)	0.1 (0.0, 0.4)

Time since diagnosis:	<1 year			1-4 years			5-9 years			10-25 years			Overall		
Cause of death	Obs	SMR (95% CI)	AER (95% CI)	Obs	SMR (95% CI)	AER (95% CI)	Obs	SMR (95% CI)	AER (95% CI)	Obs	SMR (95% CI)	AER (95% CI)	Obs	SMR (95% CI)	AER (95% CI)
Kidney cancer	15	2.19 (1.23, 3.62)	1.7 (0.3, 3.7)	20	0.94 (0.57, 1.45)	-0.1 (-0.6, 0.7)	14	0.85 (0.47, 1.43)	-0.2 (-0.9, 0.7)	13	1.28 (0.68, 2.18)	0.5 (-0.5, 1.9)	62	1.13 (0.87, 1.45)	0.2 (-0.2, 0.7)
Bladder (and other urothelial) cancer	6	0.66 (0.24, 1.45)	-0.6 (-1.4, 0.8)	27	0.89 (0.59, 1.29)	-0.2 (-0.9, 0.6)	36	1.47 (1.03, 2.03)	1.2 (0.1, 2.6)	29	1.90 (1.27, 2.73)	2.2 (0.7, 4.3)	98	1.24 (1.01, 1.51)	0.5 (0.0, 1.1)
Other genitourinary cancers	1	1.19 (0.03, 6.61)	0.0 (-0.2, 1.0)	4	1.41 (0.39, 3.62)	0.1 (-0.1, 0.5)	4	1.72 (0.47, 4.40)	0.2 (-0.1, 0.8)	3	2.01 (0.41, 5.88)	0.2 (-0.1, 1.2)	12	1.60 (0.83, 2.80)	0.1 (-0.0, 0.4)
Tumors of central nervous system and eye	53	5.91 (4.43, 7.73)	9.0 (6.3, 12.4)	55	2.03 (1.53, 2.64)	2.0 (1.0, 3.2)	31	1.53 (1.04, 2.18)	1.1 (0.1, 2.4)	16	1.26 (0.72, 2.04)	0.5 (-0.6, 2.1)	155	2.25 (1.91, 2.63)	2.5 (1.8, 3.2)
Thyroid cancer	2	3.23 (0.39, 11.67)	0.3 (-0.1, 1.4)	5	2.49 (0.81, 5.82)	0.2 (-0.0, 0.7)	4	2.52 (0.69, 6.44)	0.2 (-0.0, 0.9)	1	1.00 (0.03, 5.59)	0.0 (-0.2, 0.7)	12	2.30 (1.19, 4.02)	0.2 (0.0, 0.4)
Other endocrine gland cancer	4	18.1 (4.9, 46.2)	0.8 (0.2, 2.1)	1	1.53 (0.04, 8.53)	0.0 (-0.0, 0.3)	0	0.00 (0.00, 7.66)	0.0 (-0.0, 0.3)	1	3.43 (0.09, 19.09)	0.1 (-0.0, 0.8)	6	3.64 (1.34, 7.92)	0.1 (0.0, 0.3)
In-situ, benign or uncertain (excl hem and CNS/eye)	25	19.4 (12.6, 28.7)	4.9 (3.1, 7.3)	13	3.08 (1.64, 5.26)	0.6 (0.2, 1.3)	7	2.08 (0.84, 4.29)	0.4 (-0.1, 1.1)	1	0.50 (0.01, 2.77)	-0.2 (-0.3, 0.6)	46	4.22 (3.09, 5.63)	1.0 (0.6, 1.4)
All solid tumors	385	1.59 (1.44, 1.76)	29 (22, 38)	787	1.05 (0.97, 1.12)	2.4 (-1.4, 6.5)	791	1.37 (1.28, 1.47)	22 (16, 27)	549	1.55 (1.42, 1.68)	31 (24, 39)	2,512	1.30 (1.25, 1.36)	17 (14, 20)
Ischemic heart disease	350	2.96 (2.66, 3.29)	48 (40, 56)	394	1.11 (1.00, 1.22)	2.8 (0.1, 5.7)	324	1.26 (1.13, 1.41)	6.8 (3.3, 10.5)	180	1.23 (1.06, 1.42)	5.4 (1.3, 10.0)	1,248	1.42 (1.35, 1.50)	11 (9, 13)
Cardiomyopathy and congestive heart failure	54	4.14 (3.11, 5.41)	8.4 (5.7, 11.8)	80	1.75 (1.39, 2.18)	2.4 (1.3, 3.8)	87	2.35 (1.88, 2.90)	5.0 (3.3, 7.1)	77	3.41 (2.69, 4.26)	8.8 (6.2, 11.9)	298	2.52 (2.24, 2.82)	5.1 (4.2, 6.2)
Valvular heart disease	23	3.33 (2.11, 5.00)	3.3 (1.6, 5.7)	27	1.08 (0.71, 1.57)	0.1 (-0.5, 1.0)	43	2.03 (1.47, 2.73)	2.2 (1.0, 3.7)	28	2.13 (1.41, 3.08)	2.4 (0.9, 4.4)	121	1.83 (1.52, 2.18)	1.6 (1.0, 2.2)
Arrhythmias	19	5.08 (3.06, 7.94)	3.1 (1.6, 5.3)	32	1.94 (1.32, 2.73)	1.1 (0.4, 2.0)	25	1.57 (1.01, 2.31)	0.9 (0.0, 2.1)	28	2.61 (1.73, 3.77)	2.8 (1.3, 4.8)	104	2.21 (1.81, 2.68)	1.6 (1.1, 2.3)
Other heart	18	3.95 (2.34, 6.24)	2.8 (1.3, 4.9)	16	1.04 (0.60, 1.69)	0.0 (-0.4, 0.8)	14	1.11 (0.61, 1.86)	0.1 (-0.5, 1.1)	13	1.55 (0.82, 2.65)	0.7 (-0.2, 2.2)	61	1.49 (1.14, 1.91)	0.6 (0.2, 1.1)
All cardiac	464	3.17 (2.89, 3.47)	65 (57, 74)	549	1.20 (1.10, 1.30)	6.5 (3.3, 10.0)	493	1.43 (1.31, 1.57)	15 (11, 20)	326	1.62 (1.45, 1.81)	20 (15, 26)	1,832	1.59 (1.52, 1.67)	20 (17, 22)
Cerebrovascular	89	1.92 (1.54, 2.36)	8.8 (5.2, 13.0)	157	1.02 (0.87, 1.19)	0.2 (-1.5, 2.1)	134	1.13 (0.95, 1.34)	1.6 (-0.6, 4.1)	79	1.20 (0.95, 1.50)	2.1 (-0.5, 5.3)	459	1.19 (1.09, 1.31)	2.1 (1.0, 3.4)
Thromboembolic	62	6.51 (4.99, 8.34)	11 (8, 14)	36	1.22 (0.86, 1.69)	0.5 (-0.3, 1.5)	28	1.29 (0.85, 1.86)	0.6 (-0.3, 1.9)	9	0.72 (0.33, 1.36)	-0.6 (-1.4, 0.7)	135	1.84 (1.54, 2.18)	1.8 (1.1, 2.5)
Aortic aneurysm, peripheral vascular and other circulatory	31	1.76 (1.20, 2.50)	2.8 (0.7, 5.4)	44	0.79 (0.57, 1.06)	-0.8 (-1.7, 0.2)	37	0.87 (0.62, 1.20)	-0.5 (-1.6, 0.9)	17	0.71 (0.41, 1.13)	-1.1 (-2.3, 0.5)	129	0.92 (0.77, 1.10)	-0.3 (-0.9, 0.4)
All circulatory	646	2.94 (2.72, 3.17)	88 (77, 98)	786	1.13 (1.05, 1.21)	6.4 (2.5, 10.4)	692	1.32 (1.22, 1.42)	17 (12, 22)	431	1.42 (1.29, 1.56)	21 (14, 27)	2,555	1.46 (1.41, 1.52)	23 (20, 26)
Endocrine and metabolic diseases	45	4.17 (3.04, 5.58)	7.0 (4.5, 10.2)	44	1.21 (0.88, 1.62)	0.5 (-0.3, 1.6)	30	1.01 (0.68, 1.45)	0.0 (-0.9, 1.3)	24	1.27 (0.81, 1.89)	0.8 (-0.6, 2.7)	143	1.49 (1.26, 1.76)	1.4 (0.7, 2.1)
Dementia	12	0.42 (0.22, 0.73)	-3.4 (-4.6, -1.6)	78	0.55 (0.43, 0.68)	-4.6 (-5.8, -3.2)	145	0.99 (0.84, 1.17)	-0.1 (-2.4, 2.5)	117	1.17 (0.97, 1.40)	2.8 (-0.5, 6.5)	352	0.84 (0.76, 0.94)	-1.9 (-2.9, -0.8)
Other neurological diseases	38	1.82 (1.29, 2.50)	3.5 (1.2, 6.4)	48	0.65 (0.48, 0.87)	-1.8 (-2.7, -0.7)	64	1.05 (0.81, 1.34)	0.3 (-1.2, 2.1)	40	1.00 (0.71, 1.36)	0.0 (-1.8, 2.3)	190	0.97 (0.84, 1.12)	-0.1 (-0.9, 0.7)
COPD and asthma	108	2.22 (1.82, 2.69)	12 (8, 17)	131	0.83 (0.69, 0.98)	-2.0 (-3.5, -0.2)	141	1.12 (0.94, 1.32)	1.5 (-0.8, 4.1)	78	1.02 (0.80, 1.27)	0.2 (-2.4, 3.3)	458	1.12 (1.02, 1.22)	1.4 (0.2, 2.6)
Other respiratory diseases	72	5.58 (4.36, 7.02)	12 (9, 16)	93	2.07 (1.67, 2.54)	3.4 (2.2, 4.9)	64	1.75 (1.35, 2.24)	2.8 (1.3, 4.6)	40	1.83 (1.31, 2.50)	2.9 (1.1, 5.3)	269	2.32 (2.05, 2.61)	4.4 (3.5, 5.3)
Digestive system disease	213	5.73 (4.99, 6.56)	36 (30, 42)	172	1.42 (1.21, 1.65)	3.6 (1.9, 5.6)	129	1.36 (1.13, 1.61)	3.4 (1.3, 5.9)	99	1.68 (1.36, 2.04)	6.4 (3.4, 9.9)	613	1.96 (1.81, 2.12)	8.6 (7.2, 10.0)
Diseases of skin and subcutaneous tissue	5	2.38 (0.77, 5.55)	0.6 (-0.1, 2.0)	12	1.55 (0.80, 2.71)	0.3 (-0.1, 0.9)	11	1.67 (0.83, 2.99)	0.4 (-0.1, 1.3)	7	1.70 (0.68, 3.50)	0.5 (-0.2, 1.7)	35	1.70 (1.19, 2.37)	0.4 (0.1, 0.8)
Musculoskeletal or soft tissue disease	16	3.61 (2.06, 5.86)	2.4 (1.0, 4.4)	26	1.71 (1.11, 2.50)	0.8 (0.1, 1.6)	27	2.17 (1.43, 3.16)	1.5 (0.5, 2.7)	18	2.36 (1.40, 3.73)	1.7 (0.5, 3.4)	87	2.19 (1.75, 2.70)	1.4 (0.9, 1.9)
Kidney and urological disease	47	5.22 (3.84, 6.95)	7.8 (5.2, 11.0)	44	1.33 (0.97, 1.79)	0.8 (-0.1, 1.9)	27	0.99 (0.65, 1.43)	0.0 (-1.0, 1.2)	28	1.79 (1.19, 2.59)	2.0 (0.5, 4.0)	146	1.72 (1.45, 2.02)	1.8 (1.1, 2.5)
Other specified diseases	16	8.37 (4.78, 13.59)	2.9 (1.5, 4.9)	9	1.47 (0.67, 2.79)	0.2 (-0.1, 0.8)	6	1.28 (0.47, 2.78)	0.1 (-0.2, 0.8)	4	1.32 (0.36, 3.39)	0.2 (-0.3, 1.2)	35	2.22 (1.55, 3.09)	0.6 (0.2, 0.9)
Ill-defined diseases	5	2.29 (0.74, 5.34)	0.6 (-0.1, 1.9)	9	1.14 (0.52, 2.16)	0.1 (-0.3, 0.7)	5	0.72 (0.23, 1.67)	-0.2 (-0.5, 0.5)	7	1.40 (0.56, 2.89)	0.3 (-0.3, 1.5)	26	1.18 (0.77, 1.73)	0.1 (-0.1, 0.5)
Suicide	9	2.38 (1.09, 4.51)	1.1 (0.1, 2.7)	16	1.38 (0.79, 2.24)	0.3 (-0.2, 1.0)	6	0.71 (0.26, 1.55)	-0.2 (-0.6, 0.5)	1	0.18 (0.00, 1.00)	-0.7 (-0.9, 0.0)	32	1.09 (0.75, 1.54)	0.1 (-0.2, 0.4)
External causes (excl suicide)	73	4.64 (3.64, 5.84)	12 (9, 16)	73	1.36 (1.07, 1.72)	1.4 (0.3, 2.7)	50	1.15 (0.85, 1.51)	0.6 (-0.7, 2.3)	41	1.46 (1.05, 1.99)	2.1 (0.2, 4.5)	237	1.68 (1.48, 1.91)	2.8 (1.9, 3.7)
All causes other than hematological, solid tumor, circulatory and infection	659	3.33 (3.08, 3.59)	95 (84, 105)	755	1.06 (0.99, 1.14)	3.1 (-0.7, 7.1)	705	1.17 (1.08, 1.26)	10 (5, 16)	504	1.31 (1.20, 1.43)	19 (12, 27)	2,623	1.38 (1.33, 1.43)	21 (18, 24)

*Results for COVID-19 were based on follow-up data from 2020 onwards

†For causes that only apply to one gender, calculations were performed using the sub-cohort corresponding to that gender

Abbreviations: Obs, observed deaths; SMR, standardized mortality ratio; AER, absolute excess rate; CI, confidence interval; NA, not applicable; CNS, central nervous system; COPD, chronic obstructive pulmonary disease

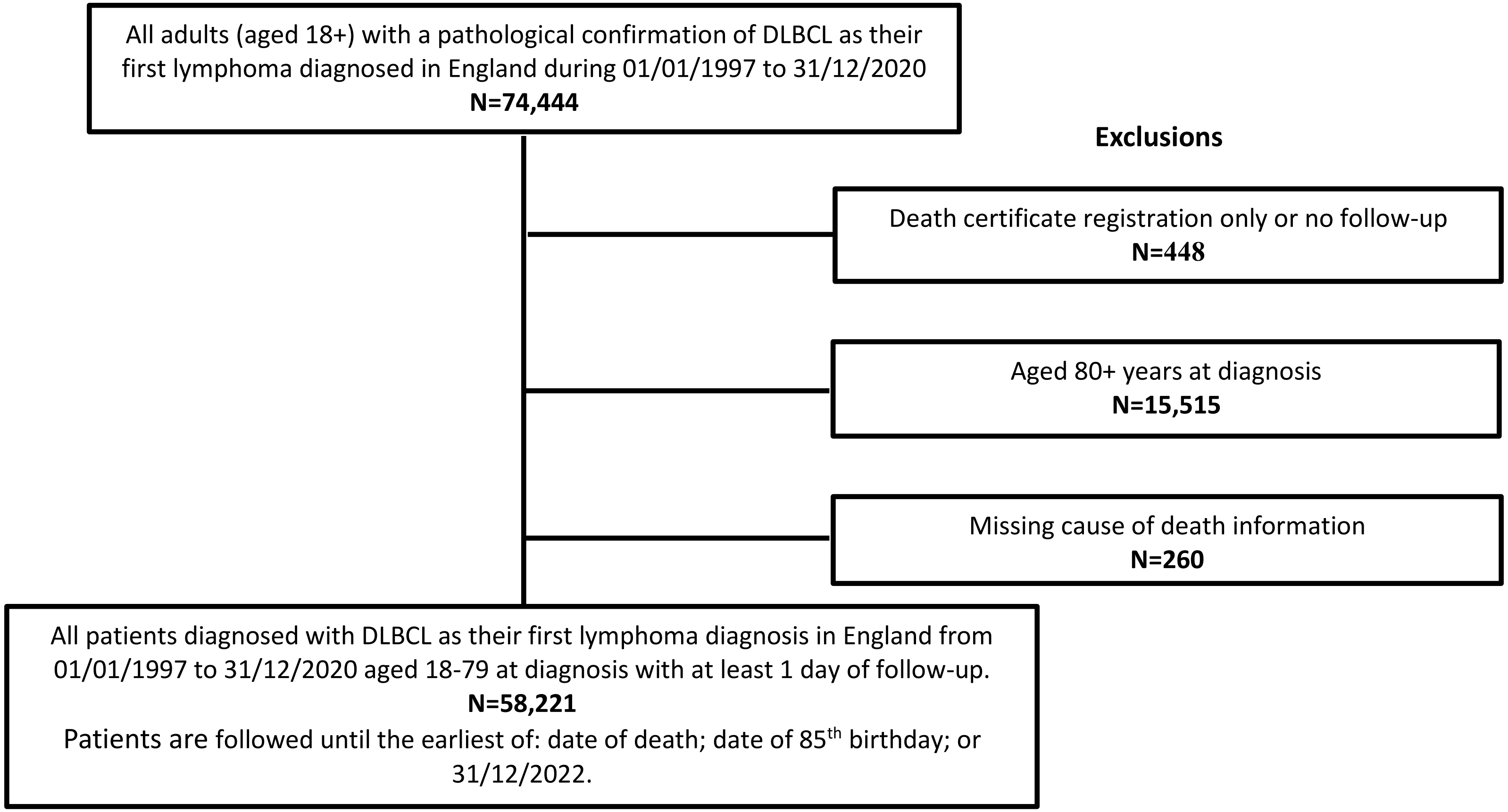

Supplemental Figure 1. Exclusion criteria for the DLBCL cohort.

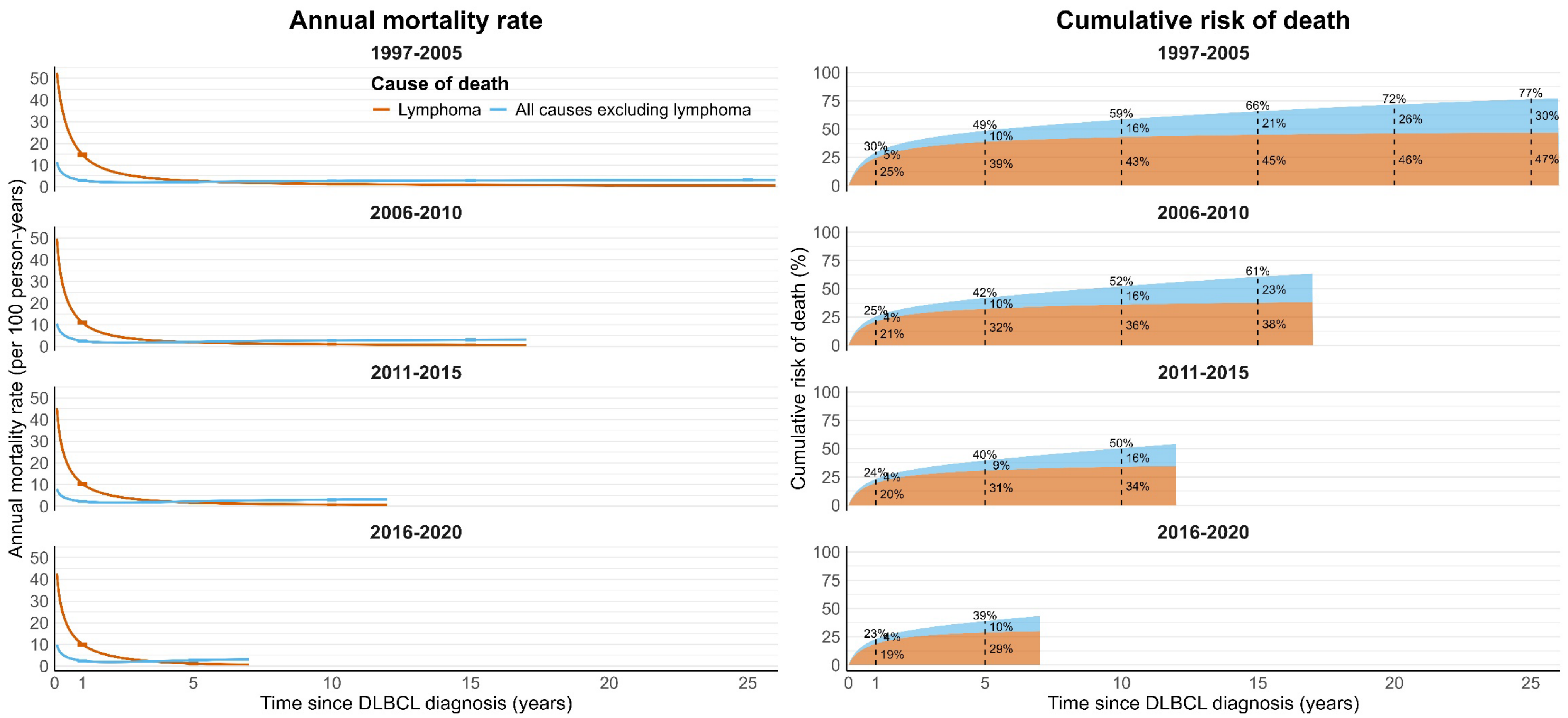

Supplemental Figure 2. Mortality rate and cumulative risk for the DLBCL cohort by time since diagnosis, calendar period of diagnosis, and cause of death. Error-bars for mortality rates indicate 95% confidence intervals. The estimates of cumulative risk for death from lymphoma are adjusted for non-lymphoma causes as a competing risk, and vice versa, so that the two risks sum to the cumulative risk of death from any cause.

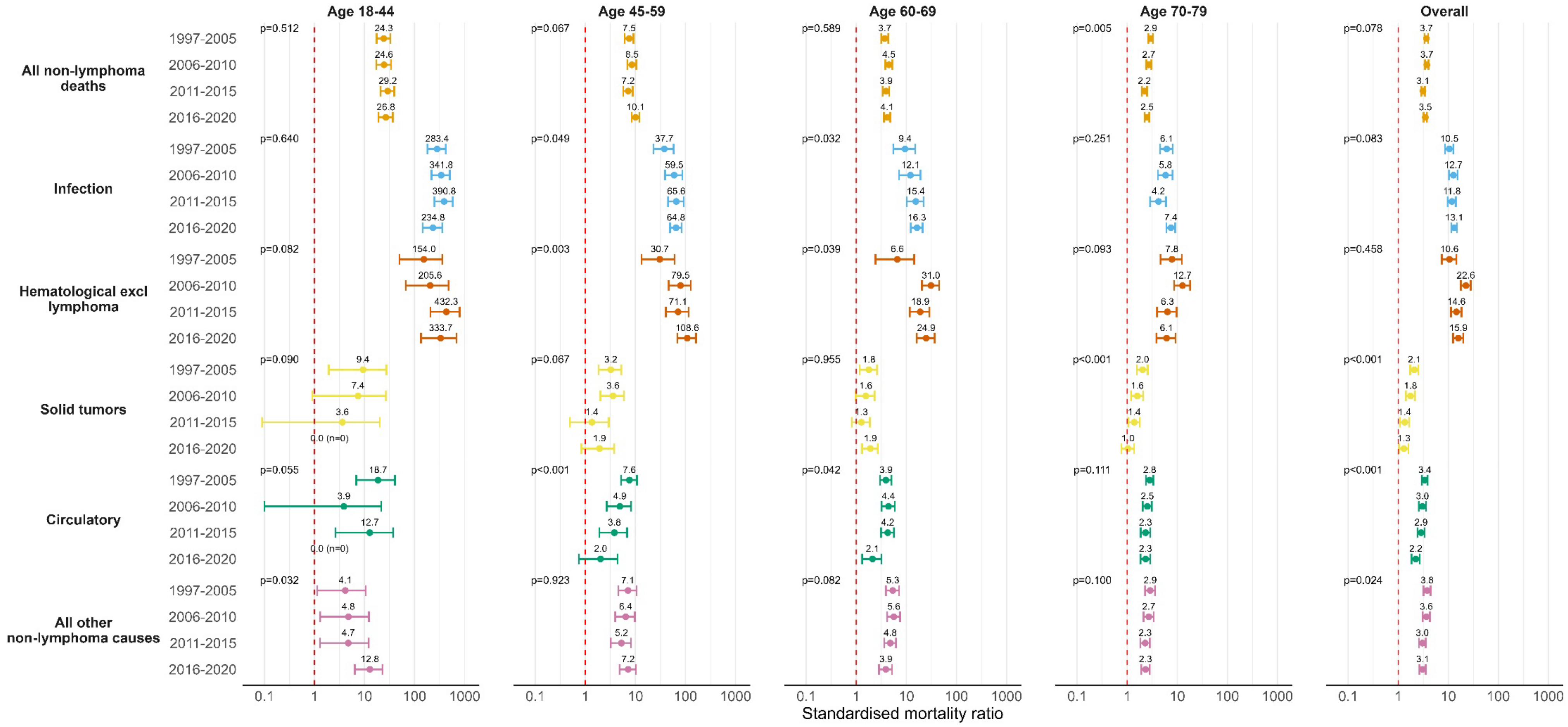

Supplemental Figure 3. Standardized mortality ratios by cause of death for the DLBCL cohort during the first year after diagnosis, by age at diagnosis and calendar period of diagnosis. Error-bars indicate 95% confidence intervals and p-values indicate tests for linear trend. Error-bars are not shown where there are no observed deaths in the cohort. Standardized mortality ratios were calculated by compared to all England rates accounting for the same calendar period, attained age, gender and deprivation quintile.

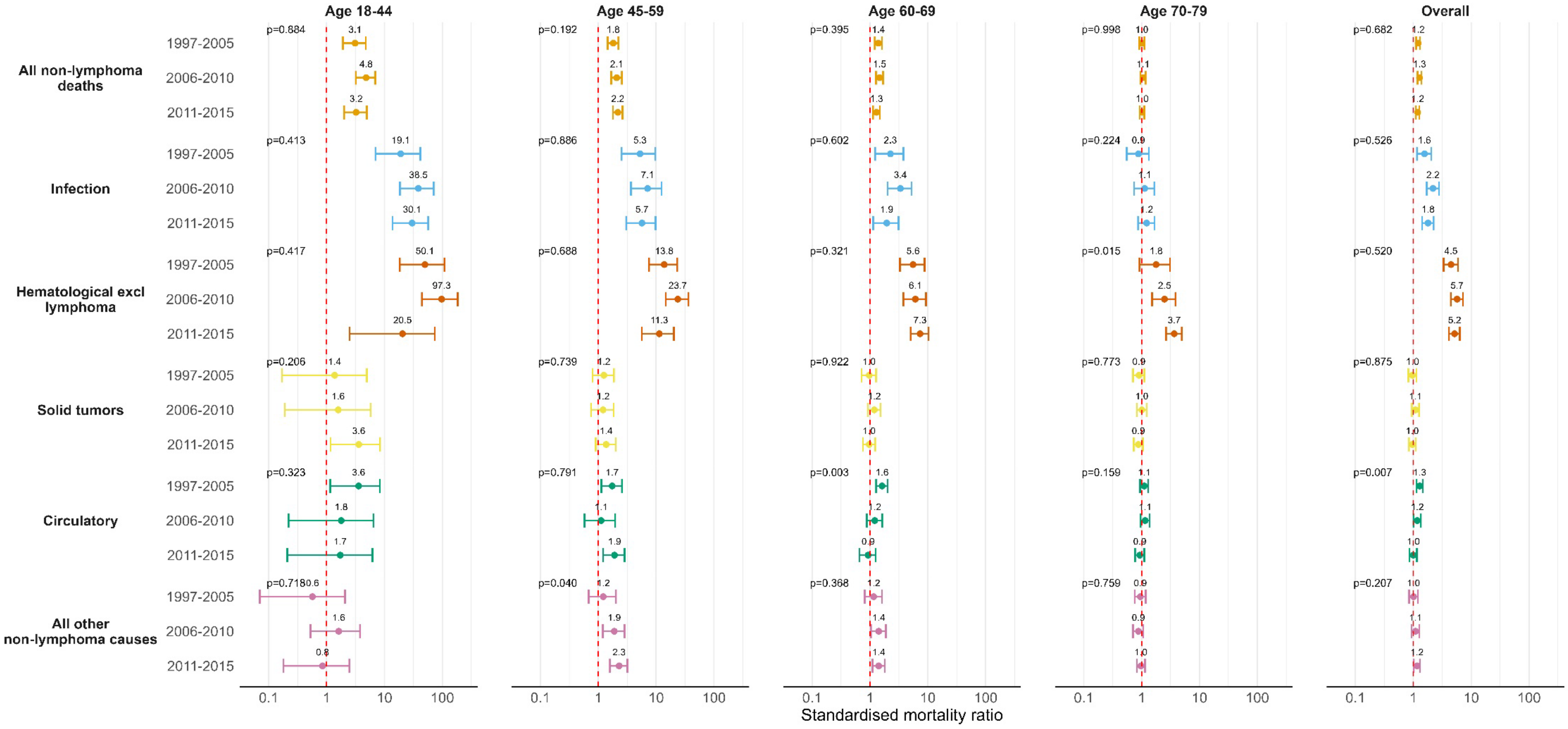

Supplemental Figure 4. Standardized mortality ratios by cause of death for the DLBCL cohort during years 1-4 after diagnosis, by age at diagnosis and calendar period of diagnosis. Error-bars indicate 95% confidence intervals and p-values indicate tests for linear trend. Standardized mortality ratios were calculated by compared to all England rates accounting for the same calendar period, attained age, gender and deprivation quintile.

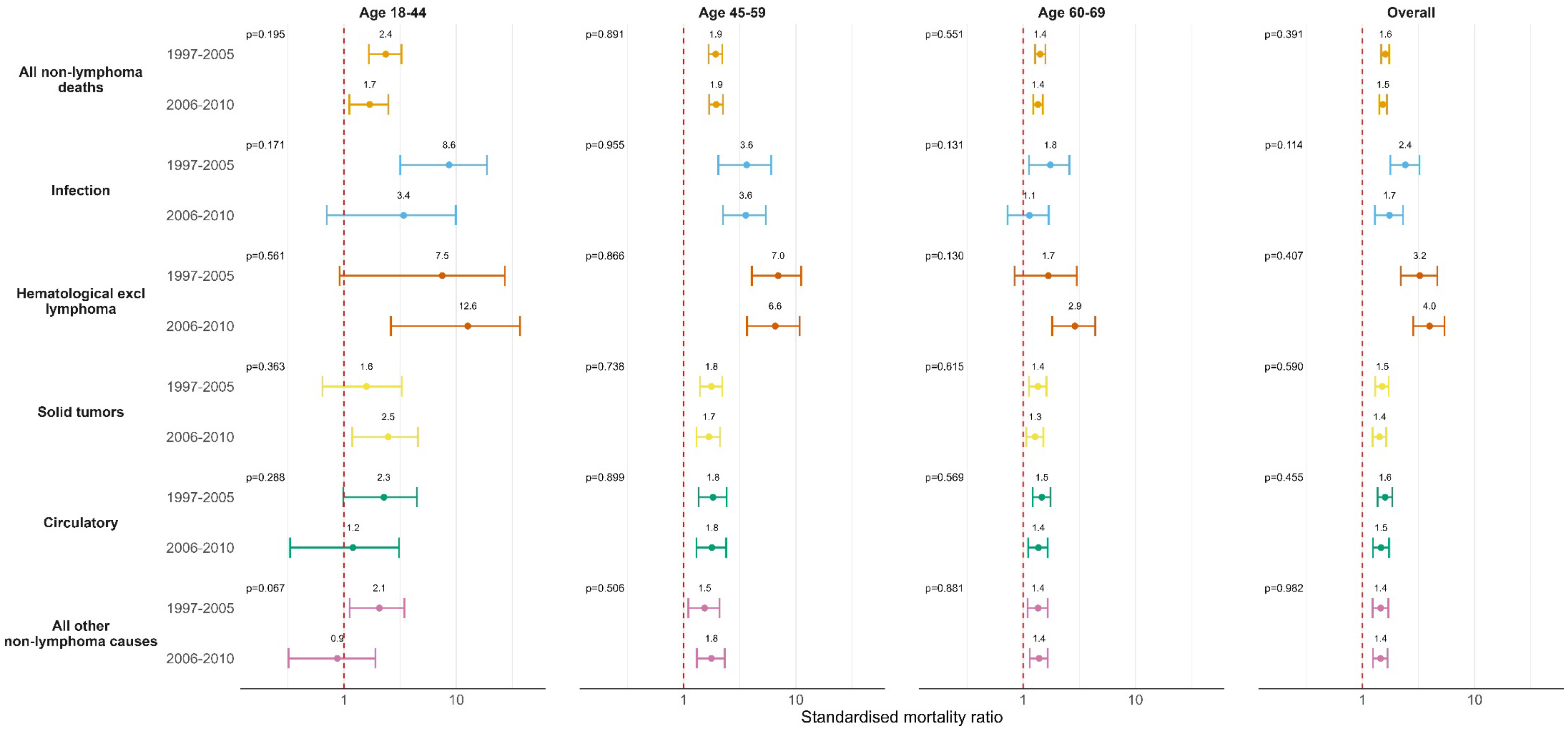

Supplemental Figure 5. Standardized mortality ratios by cause of death for the DLBCL cohort during years 5-11 after diagnosis, by age at diagnosis and calendar period of diagnosis. Error-bars indicate 95% confidence intervals and p-values indicate tests for linear trend. Standardized mortality ratios were calculated by compared to all England rates accounting for the same calendar period, attained age, gender and deprivation quintile.

Route to diagnosis: Emergency vs non-emergency

Cause of death	Rate ratio (95% CI) Emergency vs not	Excess deaths, n (%) Emergency	Non-emergency	Overall
**All causes**	2.99 (2.87, 3.13)	4723 (55)	3874 (45)	8597 (100)
**Lymphoma**	3.18 (3.03, 3.33)	NA	NA	NA
**All causes excl lymphoma**	2.19 (1.96, 2.44)	571 (56)	454 (44)	1025 (100)
All infections	1.67 (1.29, 2.17)	100 (42)	139 (58)	239 (100)
All hematological (excl lymphoma)	3.18 (2.32, 4.39)	105 (58)	77 (42)	182 (100)
All solid tumors	2.53 (1.92, 3.34)	74 (100)	-3 (0)	71 (100)
All circulatory	1.88 (1.51, 2.33)	121 (50)	119 (50)	240 (100)
All causes other than hematological, solid tumor, circulatory and infection	2.33 (1.90, 2.86)	170 (58)	122 (42)	292 (100)

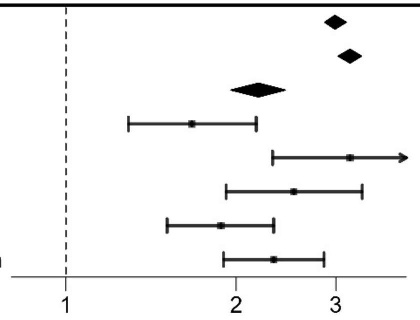

Supplemental Figure 6. Rate ratios and numbers of excess deaths by route to diagnosis (emergency vs non-emergency) for the 37,225 DLBCL patients diagnosed 2006-2018 for whom route to diagnosis was known, during the first year after diagnosis. Rate ratios are stratified by age at diagnosis, calendar period of diagnosis, gender, deprivation quintile, NHS region, and Charlson Comorbidity Index, using the categories in Table 1, as well as time since diagnosis in monthly intervals. Excess deaths are calculated by comparing to all England rates accounting for calendar period, attained age, gender and deprivation quintile.

Abbreviations: CI, confidence interval; NA, not applicable.

Age 18-59 at diagnosis

Cause of death	Rate ratio (95% CI) Emergency vs not	Excess deaths, n (%) Emergency	Non-emergency	Overall
**All causes**	3.13 (2.81, 3.48)	874 (56)	700 (44)	1574 (100)
**Lymphoma**	3.42 (3.03, 3.85)	NA	NA	NA
**All causes excl lymphoma**	2.09 (1.62, 2.69)	142 (51)	137 (49)	279 (100)
All infections	1.22 (0.80, 1.83)	41 (35)	77 (65)	118 (100)
All hematological (excl lymphoma)	2.78 (1.51, 5.15)	36 (56)	28 (44)	64 (100)
All solid tumors	2.22 (0.97, 5.11)	13 (72)	5 (28)	18 (100)
All circulatory	3.27 (1.60, 6.87)	18 (69)	8 (31)	26 (100)
All causes other than hematological, solid tumor, circulatory and infection	3.40 (1.95, 6.03)	35 (65)	19 (35)	54 (100)

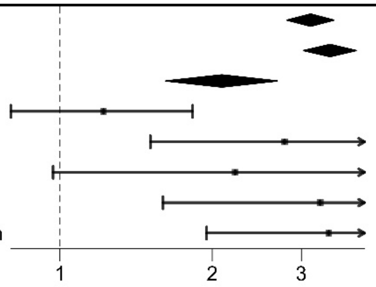

Age 60-79 at diagnosis

Cause of death	Rate ratio (95% CI) Emergency vs not	Excess deaths, n (%) Emergency	Non-emergency	Overall
**All causes**	2.97 (2.83, 3.11)	3848 (55)	3174 (45)	7022 (100)
**Lymphoma**	3.13 (2.97, 3.30)	NA	NA	NA
**All causes excl lymphoma**	2.21 (1.96, 2.49)	430 (58)	317 (42)	747 (100)
All infections	2.09 (1.49, 2.93)	60 (49)	62 (51)	122 (100)
All hematological (excl lymphoma)	3.35 (2.31, 4.89)	69 (58)	50 (42)	119 (100)
All solid tumors	2.58 (1.92, 3.46)	61 (100)	-8 (0)	53 (100)
All circulatory	1.78 (1.41, 2.23)	104 (49)	110 (51)	214 (100)
All causes other than hematological, solid tumor, circulatory and infection	2.20 (1.77, 2.74)	136 (57)	102 (43)	238 (100)

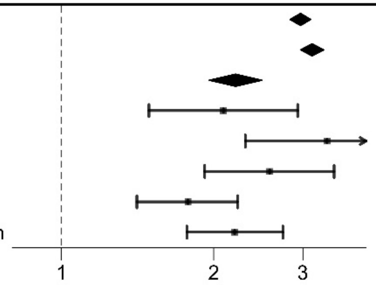

Supplemental Figure 7. Rate ratios and numbers of excess deaths by route to diagnosis (emergency vs non-emergency) for the 37,225 DLBCL patients diagnosed 2006-2018 for whom route to diagnosis was known, during the first year after diagnosis, by age at diagnosis. Rate ratios are stratified by age at diagnosis, calendar period of diagnosis, gender, deprivation quintile, NHS region, and Charlson Comorbidity Index, using the categories in Table 1, as well as time since diagnosis in monthly intervals. Excess deaths are calculated by comparing to all England rates accounting for calendar period, attained age, gender and deprivation quintile.

Abbreviations: CI, confidence interval; NA, not applicable.

Cause of death	Excess deaths n (%)
**<1 year after diagnosis**	
Infection	323 (36)
Hematological excl lymphoma	135 (15)
Solid tumors	44 (5)
Circulatory	157 (17)
All other non-lymphoma causes	243 (27)
**1-4 years after diagnosis**	
Infection	122 (43)
Hematological excl lymphoma	109 (38)
Solid tumors	23 (8)
Circulatory	2 (1)
All other non-lymphoma causes	28 (10)
**5-9 years after diagnosis**	
Infection	58 (23)
Hematological excl lymphoma	46 (18)
Solid tumors	92 (37)
Circulatory	23 (9)
All other non-lymphoma causes	34 (13)
**Overall**	
Infection	503 (35)
Hematological excl lymphoma	291 (20)
Solid tumors	159 (11)
Circulatory	182 (13)
All other non-lymphoma causes	304 (21)

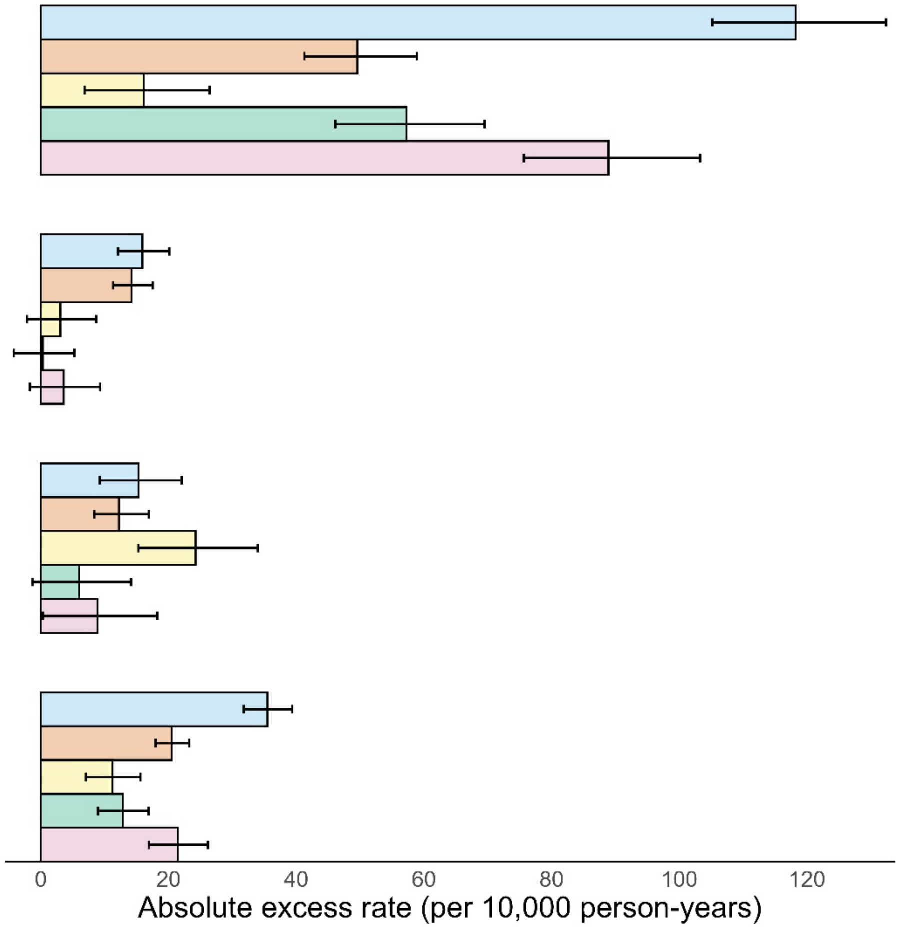

Supplemental Figure 8. Excess non-lymphoma deaths and absolute excess mortality rates for the major causes of non-lymphoma death in the DLBCL cohort in patients diagnosed from 2011, by time since diagnosis. Error-bars indicate 95% confidence intervals. Excess deaths and absolute excess rates were calculated by comparing to all England rates accounting for attained age, calendar year, gender and deprivation quintile.

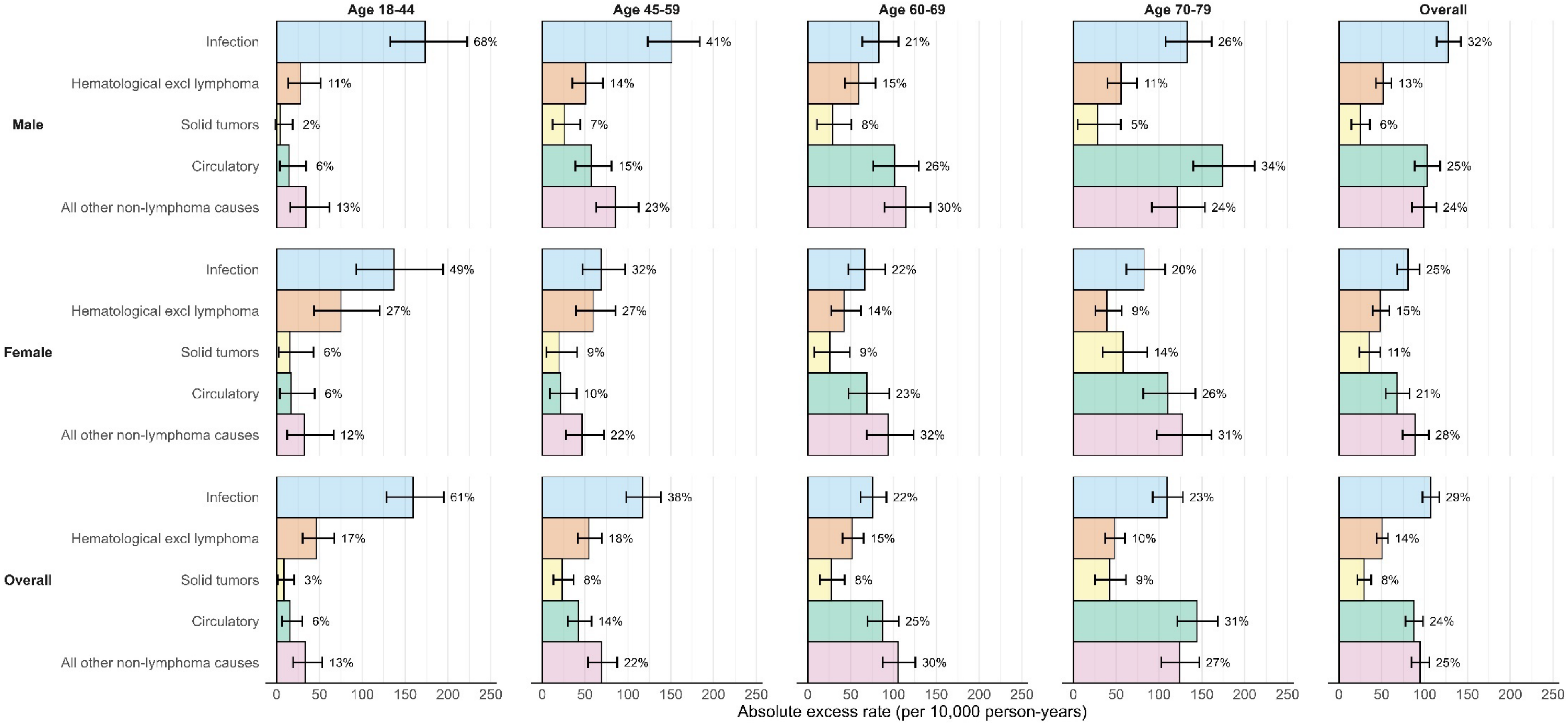

Supplemental Figure 9. Absolute excess mortality rates for the DLBCL cohort during the first year after diagnosis, by cause of death, gender and age at diagnosis. Error-bars indicate 95% confidence intervals. Percentages represent the percentage of excess non-lymphoma deaths that were attributable to that cause. Absolute excess rates were calculated by comparing to all England rates accounting for attained age, calendar year, gender and deprivation quintile.

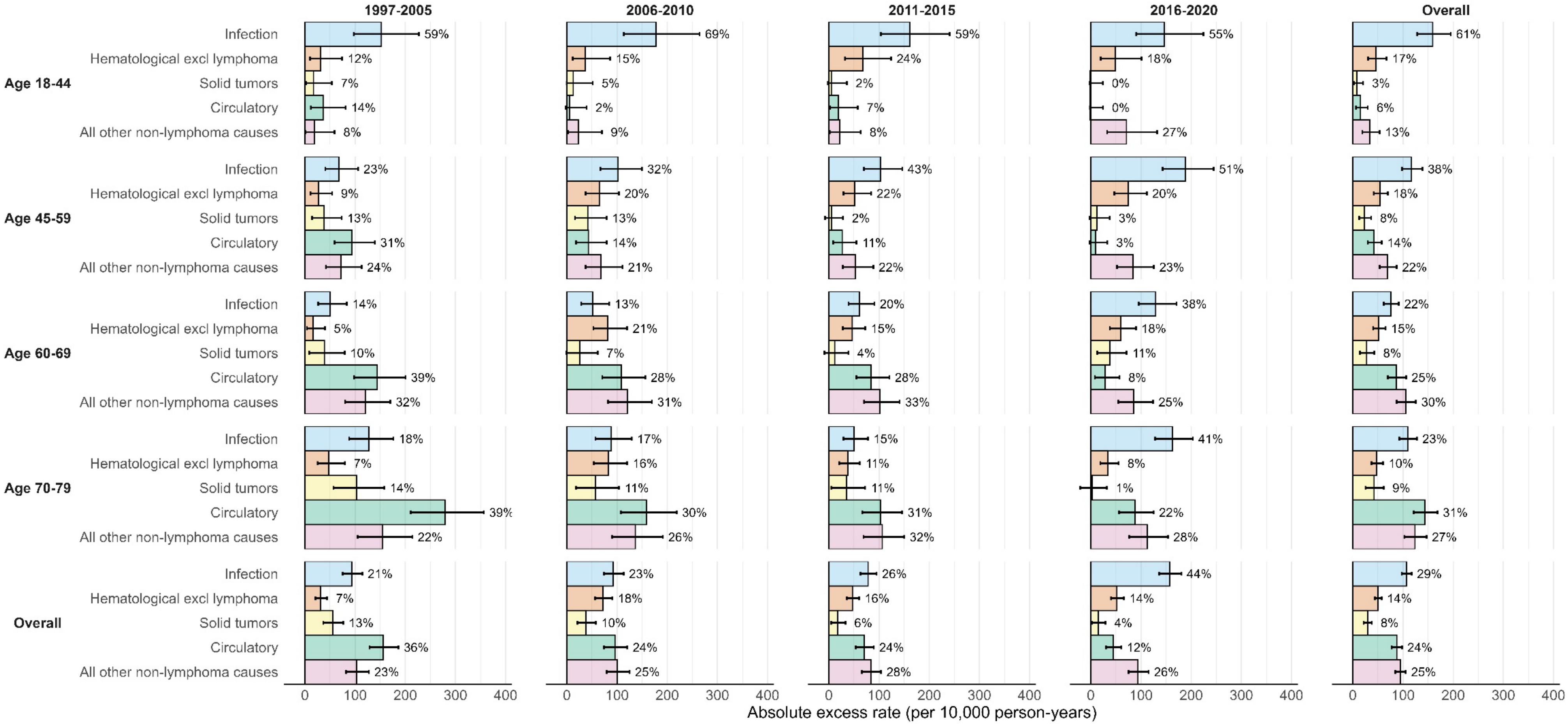

Supplemental Figure 10. Absolute excess mortality rates for the DLBCL cohort during the first year after diagnosis, by cause of death, age at diagnosis and calendar period of diagnosis. Error-bars indicate 95% confidence intervals. Percentages represent the percentage of excess non-lymphoma deaths that were attributable to that cause. Absolute excess rates were calculated by comparing to all England rates accounting for attained age, calendar year, gender and deprivation quintile.

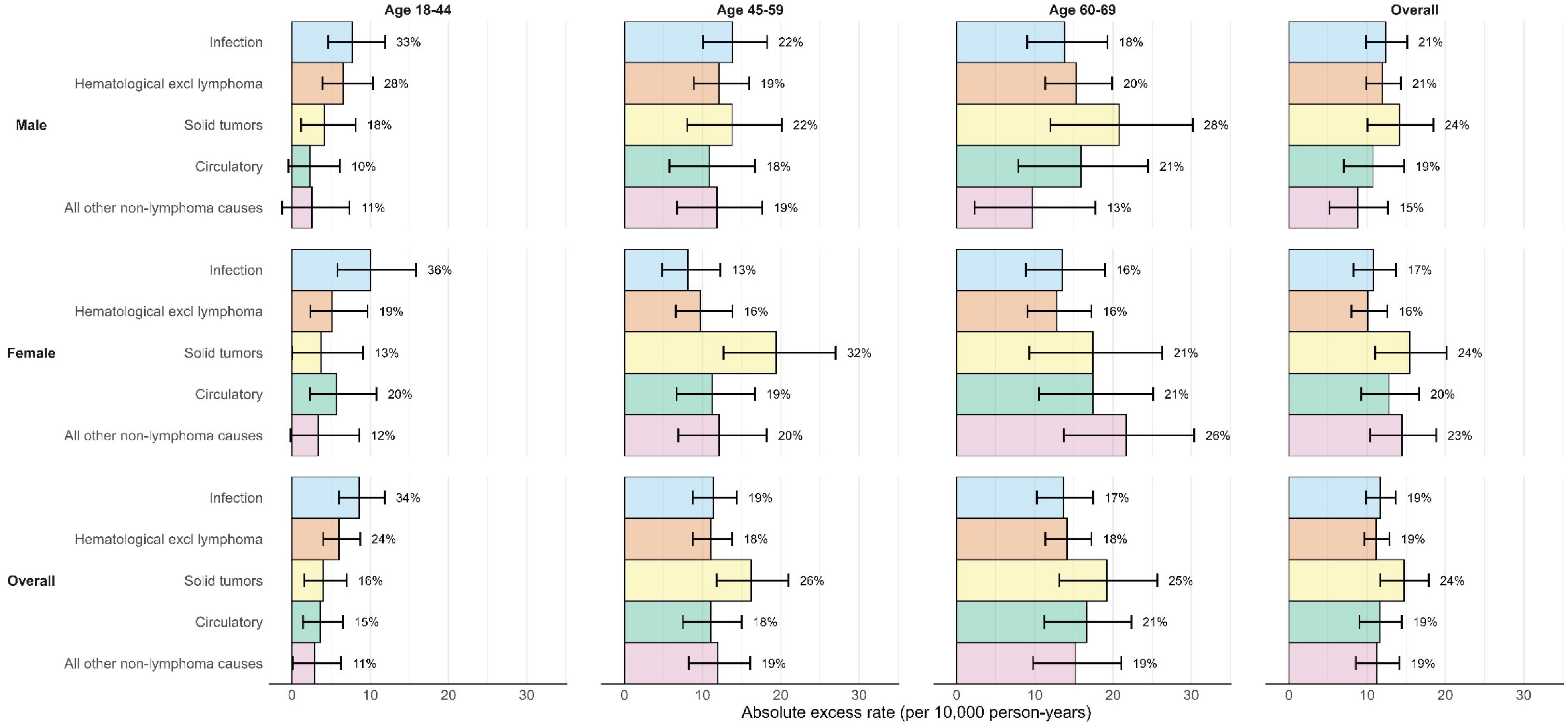

Supplemental Figure 11. Absolute excess mortality rates for the DLBCL cohort during years 1-14 after diagnosis, by cause of death, gender and age at diagnosis. Error-bars indicate 95% confidence intervals. Percentages represent the percentage of excess non-lymphoma deaths that were attributable to that cause. Absolute excess rates were calculated by comparing to all England rates accounting for attained age, calendar year, gender and deprivation quintile.

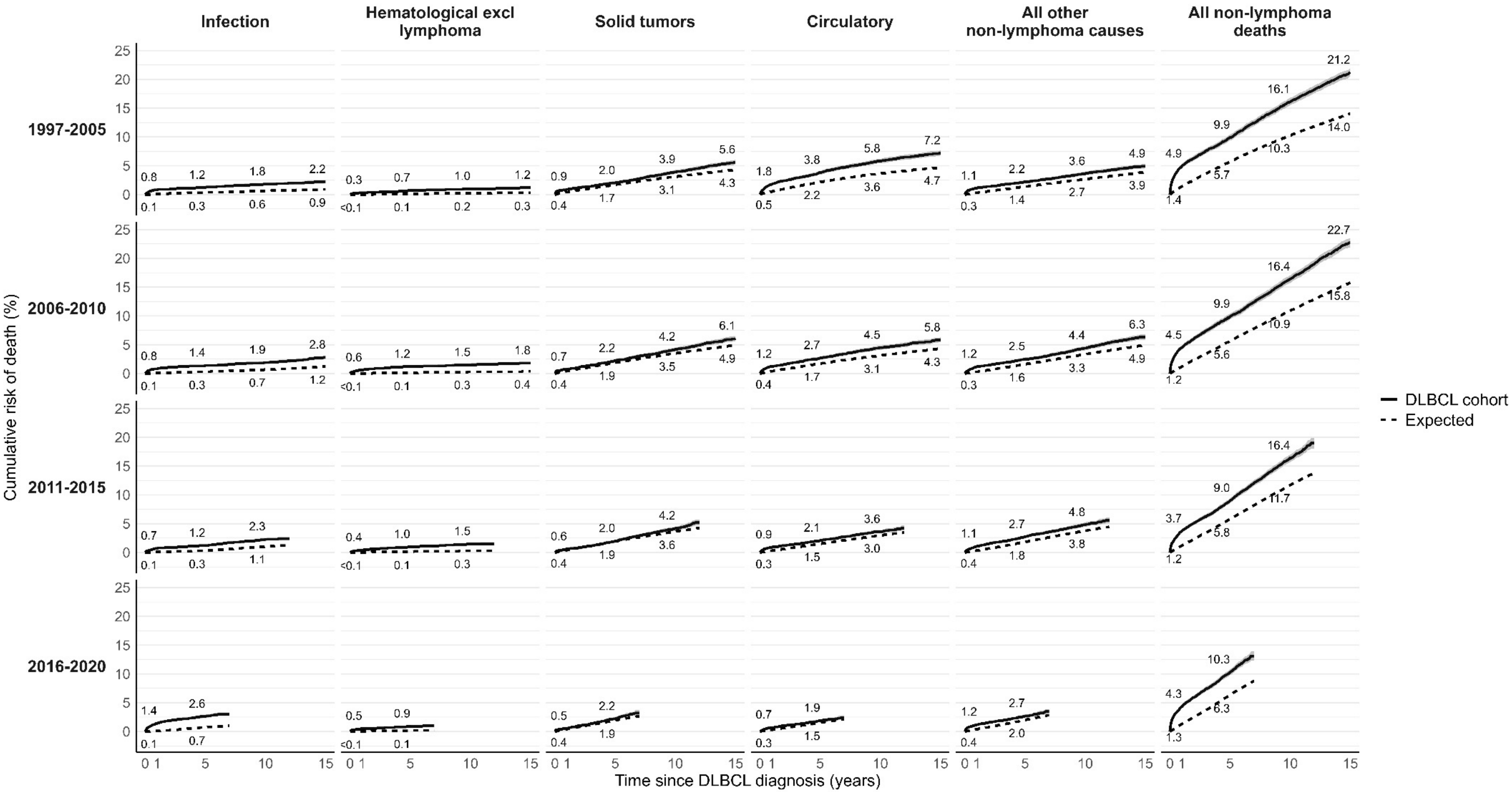

Supplemental Figure 12. Cumulative risk of death for the DLBCL cohort, by time since diagnosis, cause of death and calendar period of diagnosis. Solid lines represent the observed risk for DLBCL cohort and dashed lines the risk that would be expected based on rates for the English population, accounting for calendar period, attained age, gender and deprivation quintile. Ribbons indicate 95% confidence intervals. For each cause, both observed and expected cumulative risk estimates are adjusted for deaths from all other causes as competing risks.

Radiotherapy: Recorded vs none recorded

Cause of death	Obs	Rate ratio (95% CI) Recorded vs not	p
**All causes**	6447	0.93 (0.88, 0.99)	0.02
**Lymphoma**	2071	0.95 (0.86, 1.05)	0.35
**All causes excl lymphoma**	4376	0.92 (0.86, 0.99)	0.02
**All hematological (excl lymphoma)**	213	0.72 (0.50, 1.00)	0.06
**All infections**	491	0.89 (0.71, 1.09)	0.27
All gastrointestinal cancers	453	0.92 (0.74, 1.14)	0.46
Oesophagus cancer	83	1.09 (0.65, 1.78)	0.72
Stomach cancer	32	0.83 (0.34, 1.83)	0.66
Colorectal cancer	137	0.85 (0.56, 1.26)	0.43
Pancreas cancer	100	1.04 (0.64, 1.64)	0.86
Other gastrointestinal cancer	101	0.81 (0.49, 1.28)	0.38
Head and neck cancers	49	1.91 (1.03, 3.44)	0.03
Lung cancer	385	0.87 (0.68, 1.10)	0.24
Cancer of soft tissue, bones and joints	32	1.28 (0.58, 2.71)	0.52
Melanoma of skin	33	0.51 (0.19, 1.20)	0.15
Female breast cancer	70	0.44 (0.20, 0.86)	0.03
Cervix and uterine cancer	20	1.54 (0.53, 3.97)	0.39
Cancer of ovary or fallopian tubes	25	0.41 (0.10, 1.22)	0.16
Prostate cancer	90	1.19 (0.74, 1.88)	0.46
Kidney cancer	27	0.55 (0.18, 1.37)	0.24
Bladder (and other urothelial) cancer	65	0.87 (0.48, 1.52)	0.65
Tumors of central nervous system and eye	47	1.25 (0.63, 2.33)	0.51
**All solid tumors**	1340	0.91 (0.80, 1.03)	0.15
Ischemic heart disease	504	0.84 (0.67, 1.04)	0.11
Cardiomyopathy and congestive heart failure	164	0.51 (0.33, 0.76)	0.002
Valvular heart disease	71	0.74 (0.40, 1.31)	0.32
Arrhythmias	53	0.48 (0.21, 0.96)	0.05
Other heart	27	1.43 (0.57, 3.31)	0.42
**All cardiac**	819	0.74 (0.62, 0.88)	<0.001
Cerebrovascular	213	1.22 (0.89, 1.65)	0.21
Thromboembolic	37	1.16 (0.54, 2.36)	0.68
Aortic aneurysm, peripheral vascular and other circulatory	54	1.08 (0.57, 1.97)	0.80
**All circulatory**	1123	0.85 (0.74, 0.98)	0.03
**All causes other than hematological, solid tumor, circulatory and infection**	1209	1.06 (0.93, 1.21)	0.40

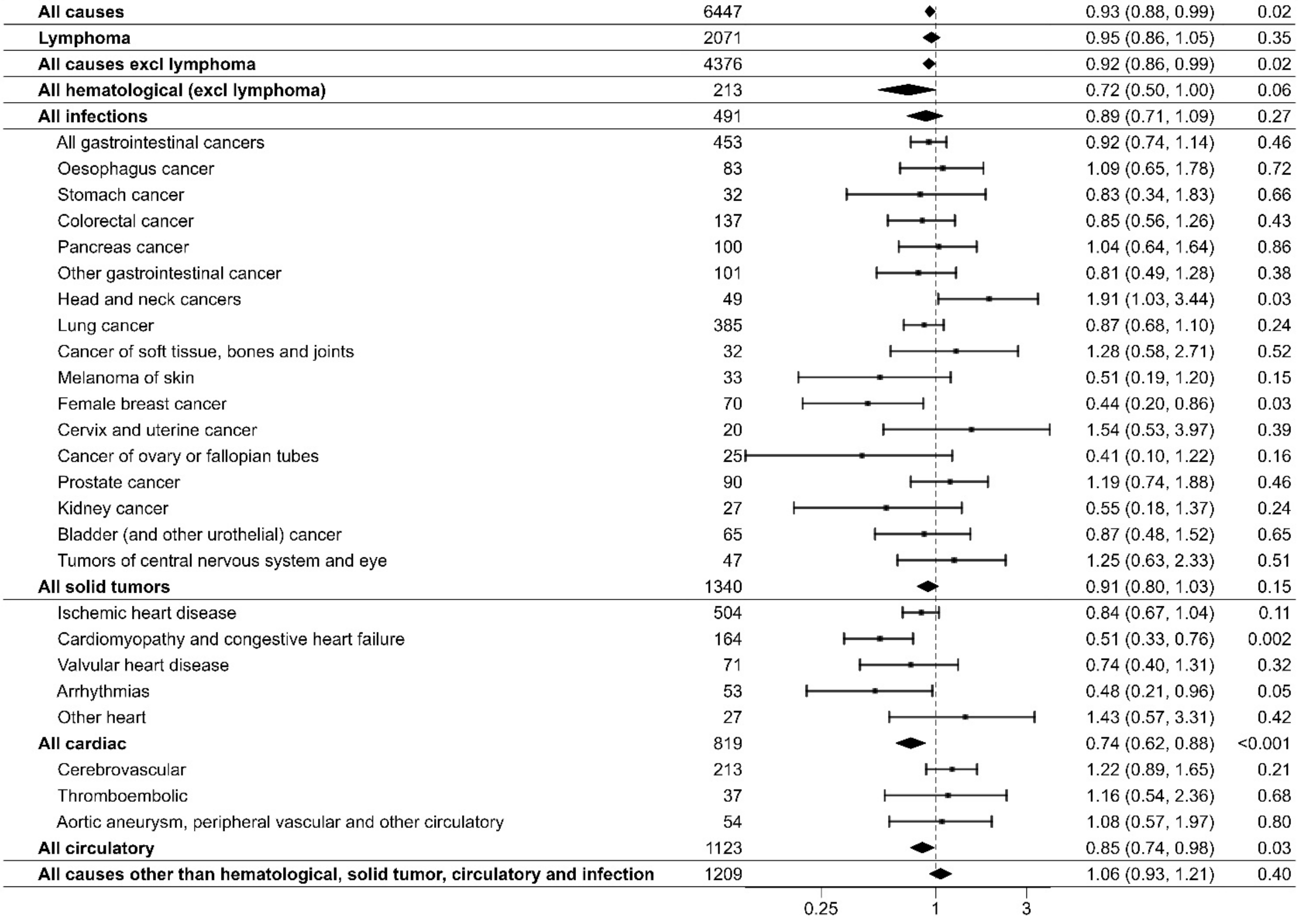

Supplemental Figure 13. Rate ratios by recorded use of radiotherapy for the whole DLBCL cohort from 5 years after diagnosis. Individual causes are shown for individual types of solid tumors and circulatory diseases if there were at least 20 observed deaths. Rate ratios are stratified by age at diagnosis, calendar period of diagnosis, gender, deprivation quintile, and NHS region, using the categories in Table 1, as well as time since diagnosis in yearly intervals.

Abbreviations: Obs, observed deaths; CI, confidence interval.

Radiotherapy: Recorded vs none recorded

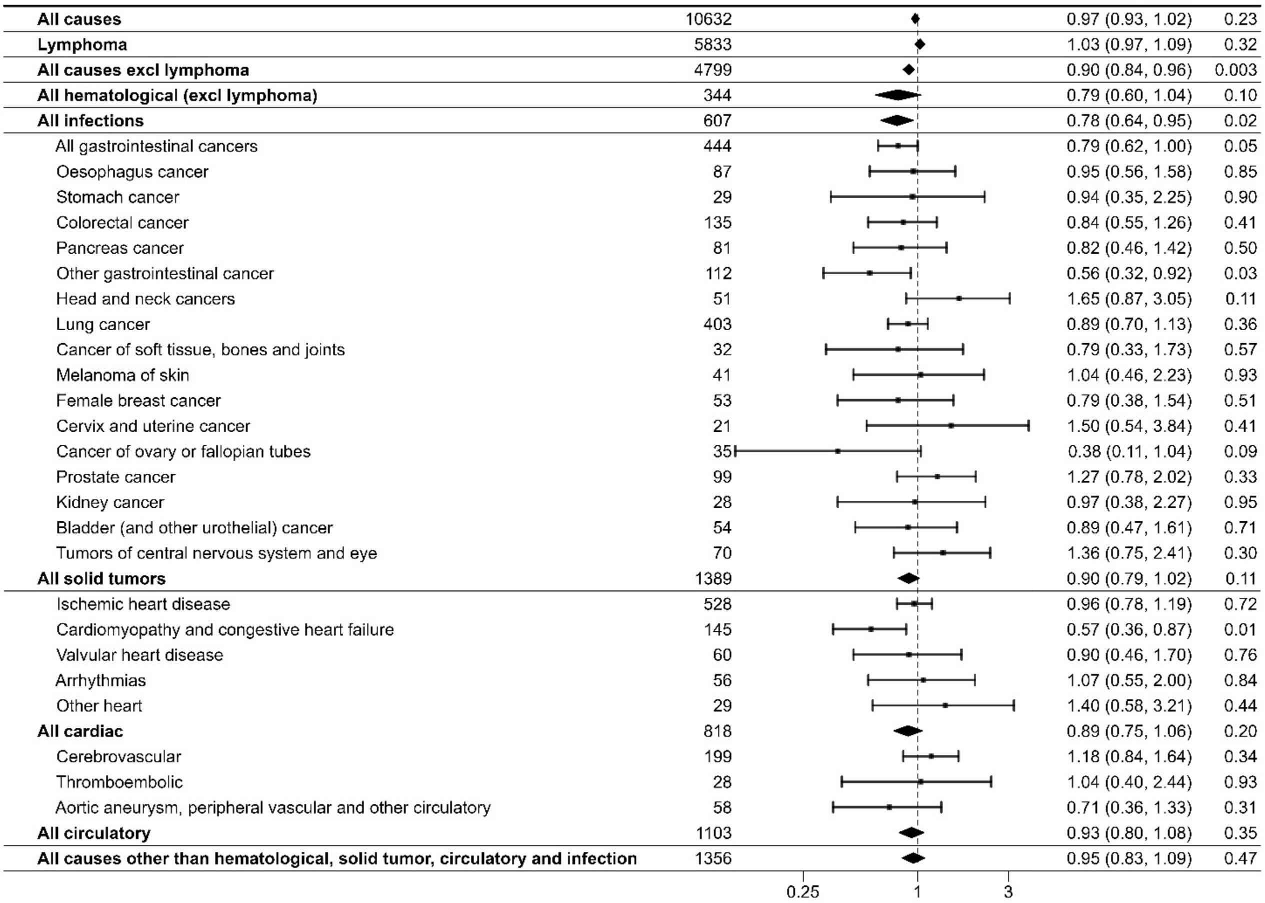

Cause of death	Obs	Rate ratio (95% CI) Recorded vs not	p
**All causes**	10632	0.97 (0.93, 1.02)	0.23
**Lymphoma**	5833	1.03 (0.97, 1.09)	0.32
**All causes excl lymphoma**	4799	0.90 (0.84, 0.96)	0.003
**All hematological (excl lymphoma)**	344	0.79 (0.60, 1.04)	0.10
**All infections**	607	0.78 (0.64, 0.95)	0.02
All gastrointestinal cancers	444	0.79 (0.62, 1.00)	0.05
Oesophagus cancer	87	0.95 (0.56, 1.58)	0.85
Stomach cancer	29	0.94 (0.35, 2.25)	0.90
Colorectal cancer	135	0.84 (0.55, 1.26)	0.41
Pancreas cancer	81	0.82 (0.46, 1.42)	0.50
Other gastrointestinal cancer	112	0.56 (0.32, 0.92)	0.03
Head and neck cancers	51	1.65 (0.87, 3.05)	0.11
Lung cancer	403	0.89 (0.70, 1.13)	0.36
Cancer of soft tissue, bones and joints	32	0.79 (0.33, 1.73)	0.57
Melanoma of skin	41	1.04 (0.46, 2.23)	0.93
Female breast cancer	53	0.79 (0.38, 1.54)	0.51
Cervix and uterine cancer	21	1.50 (0.54, 3.84)	0.41
Cancer of ovary or fallopian tubes	35	0.38 (0.11, 1.04)	0.09
Prostate cancer	99	1.27 (0.78, 2.02)	0.33
Kidney cancer	28	0.97 (0.38, 2.27)	0.95
Bladder (and other urothelial) cancer	54	0.89 (0.47, 1.61)	0.71
Tumors of central nervous system and eye	70	1.36 (0.75, 2.41)	0.30
**All solid tumors**	1389	0.90 (0.79, 1.02)	0.11
Ischemic heart disease	528	0.96 (0.78, 1.19)	0.72
Cardiomyopathy and congestive heart failure	145	0.57 (0.36, 0.87)	0.01
Valvular heart disease	60	0.90 (0.46, 1.70)	0.76
Arrhythmias	56	1.07 (0.55, 2.00)	0.84
Other heart	29	1.40 (0.58, 3.21)	0.44
**All cardiac**	818	0.89 (0.75, 1.06)	0.20
Cerebrovascular	199	1.18 (0.84, 1.64)	0.34
Thromboembolic	28	1.04 (0.40, 2.44)	0.93
Aortic aneurysm, peripheral vascular and other circulatory	58	0.71 (0.36, 1.33)	0.31
**All circulatory**	1103	0.93 (0.80, 1.08)	0.35
**All causes other than hematological, solid tumor, circulatory and infection**	1356	0.95 (0.83, 1.09)	0.47

Supplemental Figure 14. Rate ratios by recorded use of radiotherapy for the 45,025 DLBCL patients diagnosed 2006 onwards, from 1 year after diagnosis. Individual causes are shown for individual types of solid tumors and circulatory diseases if there were at least 20 observed deaths. Rate ratios are stratified by age at diagnosis, calendar period of diagnosis, gender, deprivation quintile, Charlson Comorbidity Index, and NHS region, using the categories in Table 1, as well as time since diagnosis in yearly intervals.

Abbreviations: Obs, observed deaths; CI, confidence interval.

Radiotherapy: Recorded vs none recorded

Cause of death	Obs	Rate ratio (95% CI) Recorded vs not	p
**All causes**	3663	0.94 (0.85, 1.04)	0.22
**Lymphoma**	1155	0.95 (0.80, 1.13)	0.58
**All causes excl lymphoma**	2508	0.93 (0.82, 1.05)	0.26
**All hematological (excl lymphoma)**	133	0.59 (0.30, 1.07)	0.10
**All infections**	301	0.82 (0.57, 1.16)	0.28
All gastrointestinal cancers	240	0.81 (0.52, 1.22)	0.33
Oesophagus cancer	49	0.69 (0.23, 1.72)	0.47
Colorectal cancer	72	0.98 (0.47, 1.87)	0.96
Pancreas cancer	48	0.43 (0.10, 1.28)	0.18
Other gastrointestinal cancer	56	0.73 (0.27, 1.67)	0.50
Head and neck cancers	27	1.35 (0.43, 3.58)	0.58
Lung cancer	231	0.96 (0.63, 1.43)	0.86
Melanoma of skin	20	0.88 (0.13, 3.59)	0.88
Female breast cancer	33	1.10 (0.36, 2.73)	0.85
Prostate cancer	58	0.64 (0.22, 1.52)	0.36
Bladder (and other urothelial) cancer	33	1.01 (0.37, 2.38)	0.99
Tumors of central nervous system and eye	31	0.99 (0.32, 2.55)	0.99
**All solid tumors**	758	0.94 (0.74, 1.16)	0.56
Ischemic heart disease	270	1.02 (0.68, 1.47)	0.94
Cardiomyopathy and congestive heart failure	87	0.24 (0.07, 0.59)	0.006
Valvular heart disease	38	0.38 (0.06, 1.33)	0.20
Arrhythmias	29	0.50 (0.08, 1.88)	0.37
**All cardiac**	439	0.72 (0.51, 0.99)	0.05
Cerebrovascular	100	0.68 (0.34, 1.26)	0.24
Aortic aneurysm, peripheral vascular and other circulatory	28	0.16 (0.01, 0.83)	0.08
**All circulatory**	579	0.70 (0.52, 0.92)	0.01
**All causes other than hematological, solid tumor, circulatory and infection**	737	1.26 (1.01, 1.55)	0.03

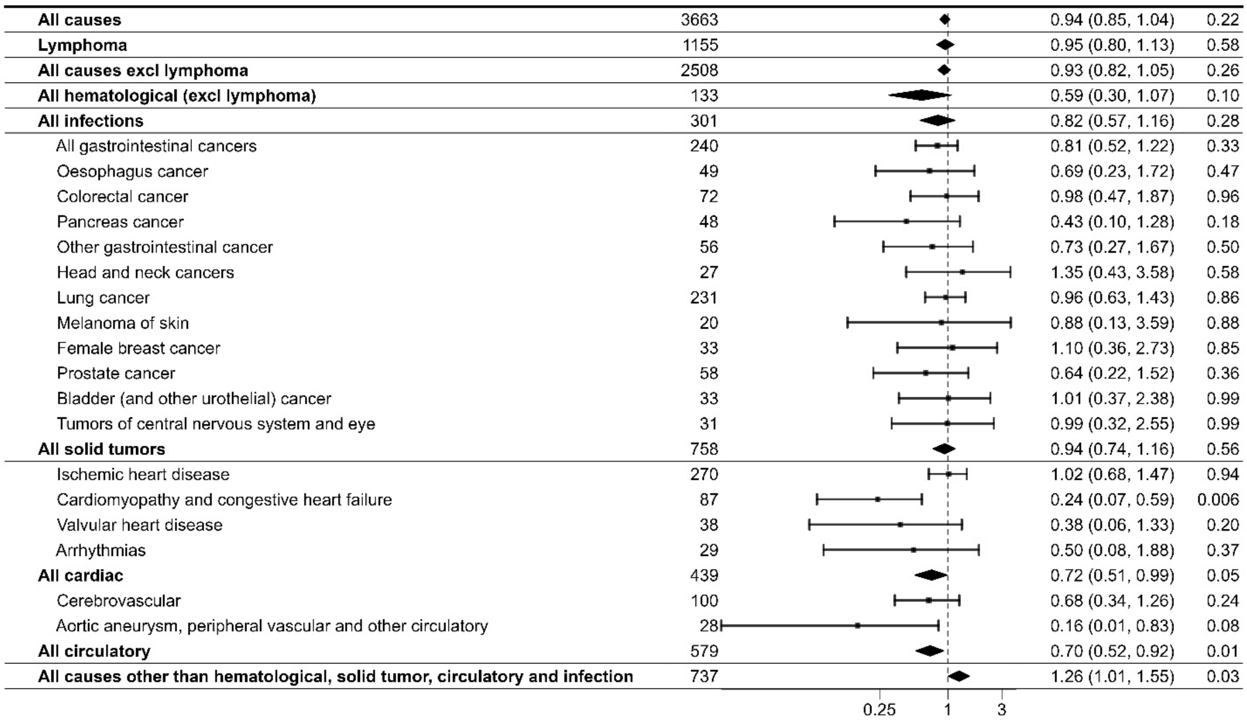

Supplemental Figure 15. Rate ratios by recorded use of radiotherapy for the 45,025 DLBCL patients diagnosed 2006 onwards, from 5 years after diagnosis. Individual causes are shown for individual types of solid tumors and circulatory diseases if there were at least 20 observed deaths. Rate ratios are stratified by age at diagnosis, calendar period of diagnosis, gender, deprivation quintile, Charlson Comorbidity Index, and NHS region, using the categories in Table 1, as well as time since diagnosis in yearly intervals. In this analysis, recorded radiotherapy was counted for up to two years post-DLCBL diagnosis, as opposed to one year in Figure 4.

Abbreviations: Obs, observed deaths; CI, confidence interval.
